# Non-fatal overdose risk during and after opioid agonist treatment: A primary care cohort study with linked hospitalisation and mortality records

**DOI:** 10.1016/j.lanepe.2022.100489

**Published:** 2022-08-11

**Authors:** Eleni Domzaridou, Matthew J. Carr, Roger T. Webb, Tim Millar, Darren M. Ashcroft

**Affiliations:** a National Institute for Health and Care Research Greater Manchester Patient Safety Translational Research Centre, Manchester Academic Health Science Centre, University of Manchester, UK; b Centre for Pharmacoepidemiology and Drug Safety, Division of Pharmacy and Optometry, School of Health Sciences, Faculty of Biology, Medicine and Health, University of Manchester, UK; c Centre for Mental Health and Safety, Division of Psychology and Mental Health, School of Health Sciences, Faculty of Biology, Medicine and Health, University of Manchester, UK

**Keywords:** Opioid use disorder, Overdose, Methadone, Buprenorphine, Drug safety, CPRD

## Abstract

**Background:**

The initiation and cessation of opioid agonist treatment (OAT) have both been associated with elevated risk of fatal overdose. We examined risk of non-fatal overdose during OAT initiation and cessation and specifically between methadone versus buprenorphine recipients.

**Methods:**

We utilised primary care electronic health records from the Clinical Practice Research Datalink to delineate a study cohort of adults aged 18-64 who were prescribed OAT between Jan 1, 1998 and Dec 31, 2017. These records were linked to hospitalisation, mortality records and patient neighbourhood and practice-level Index of Multiple Deprivation quintiles. With inverse probability treatment weights applied and negative binomial regression models we estimated incidence rate ratios for hospital admissions among patients who experienced multiple overdoses.

**Findings:**

A total of 20898 patients were prescribed methadone or buprenorphine over 83856 person-years of follow-up. Compared with periods in treatment, patients not in treatment were 51% more likely to experience a non-fatal overdose that required hospitalisation (weighted rate ratio, wRR 1·51; 95% CI 1·42, 1·60), especially during the four weeks of OAT initiation (5·59; 5·31, 5·89) and following cessation (13·39; 12·78, 14·03). The wRR of overdose during (0·37; 0·34, 0·39) and after treatment (0·36; 0·34, 0·38) favoured buprenorphine compared to methadone.

**Interpretation:**

OAT is associated with decreased non-fatal overdose risk. Buprenorphine may act more protectively than methadone, especially during the first four weeks of treatment.

**Funding:**

National Institute for Health and Care Research (NIHR) Greater Manchester Patient Safety Translational Research Centre (PSTRC-2016-003).

**Research in context**

**Evidence before this study**

We searched PubMed from database inception to Dec 10, 2021. We applied combinations of search terms related to overdose (“overdos*” or “poison*” AND “nonfatal” or “non-fatal” or “non fatal” or “accidental”) and opioid agonist treatment (methadone or buprenorphine or “substitution therapy” or “substitution treatment”) in titles or abstracts. The search yielded 339 studies. We excluded 334 articles because they were not conducted in human populations, did not examine overdose as an outcome or were not relevant to substance misuse. From the five studies that we reviewed, one cohort study examined the effectiveness of OAT compared to other interventions or no pharmacological treatment and found that patients who received OAT were less likely to experience opioid-related overdose or use acute care for serious overdose complications. Another cohort study showed that buprenorphine is associated with shorter length of stay due to opioid overdose than methadone. One study examined the risk of accidental overdose among methadone and buprenorphine recipients. By implementing the within-individual study design, these investigators found an elevated risk among methadone recipients. In a self-controlled case-series study that examined treatment time intervals and the risk of overdose, patients during OAT had a lower risk of overdose compared to those who ceased treatment.

**Added value of this study**

Utilising data from a cohort of 20898 patients receiving OAT in primary care in England, we investigated potential fluctuations in risk of hospitalisation due to non-fatal overdose by examining different periods in and out of treatment over 83856 years of follow-up in aggregate. We estimated both incidence rates and event rates (the latter including multiple overdoses per patient) for non-fatal overdose. Compared with periods out of treatment, patients who were prescribed OAT were less likely to experience a non-fatal overdose resulting in hospitalisation. Moreover, non-fatal overdose risk was elevated during the first four weeks in treatment compared to the remaining period through which patients remained in treatment. We also found that buprenorphine recipients had an overall lower overdose risk compared to methadone recipients. Buprenorphine may be more effective compared to methadone during treatment initiation and after treatment cessation.

**Implications of all the available evidence**

Non-fatal overdose is known to be a strong risk factor for subsequent fatal overdose. Our findings enhance the understanding of overdose risk on treatment initiation and cessation and in relation to methadone versus buprenorphine treatment. Currently, the evidence-base informing medication choices for opioid use disorder is incomplete. Furthermore, only few patients engage with treatment services and medication following hospital admission due to drug overdose. Internationally, the surveillance system for non-fatal overdoses is less developed compared to that of fatal overdoses and secondary care can play an important role in identifying patients at elevated overdose risk.

Alt-text: Unlabelled box

## Introduction

Overdose is one of the most serious adverse events among people who misuse opioids. Fatal overdoses, especially opioid-related ones, have attracted considerable attention worldwide, with the Global Burden of Disease study estimating that opioids were responsible for more than 65% of all drug-related deaths that occurred in 2017.^1^ Non-fatal overdose is associated with an increased risk of overdose repetition,^2^ morbidity,^3^ and fatal overdose^4^ and is much more common than fatal overdose. Colledge et al.^5^ in a systematic review of 75 studies that were conducted between 2002 and 2017, estimated that around a fifth people who injected drugs were likely to die from overdose. Whereas 20·5% (95% CI 15·0, 26·1%) of people who injected drugs experienced overdose over one year of follow-up, 41·5% (95% CI 34·6, 48·4%) experienced it during their lifetime.^5^

Fatal overdose risk during and after treatment provision has been widely studied,^6^ and may differ by treatment modality,^7^ age,^8^ and health status,^9^ but little is known about the risk of hospitalisation due to overdose. Although sustained treatment with opioid agonists is associated with reduced non-fatal overdose risk compared to no treatment or non-pharmacological interventions,^10^ the potential association between different pharmacotherapies and overdose risk, short- and long-term, is incompletely understood.

According to the World Health Organization, methadone and buprenorphine are essential medicines, and therefore conducting randomised trials with an effective treatment withheld would be unethical. Although drug overdose is among the most common diagnoses at hospital discharge and is the predominant cause of death among people with opioid use disorder,^3^ little is known about the potential protection that different opioid agonist treatment (OAT) modalities provide against non-fatal overdoses. As a full agonist, methadone may carry higher overdose risk, especially during titration that involves rapid dosage increase. The half-life of methadone varies between 8 and 59 hours, and may lead to a prolonged period of respiratory depression.^11^ The partial agonist buprenorphine is theoretically safer because of its lower respiratory depression risk due to its ceiling effect, although concomitant use of other drugs and substances may compromise its relative safety.

In the UK, methadone and buprenorphine are recommended pharmacotherapies, although guidelines pertaining to medication choice are limited.^12^ Therefore, patients’ or prescribers’ preferences tend to determine treatment decisions, with methadone often the first-line treatment in the absence of contraindications. However, in other countries, buprenorphine is suggested as first-line pharmacotherapy for opioid use disorder.^13^^,^^14^

The primary purpose of this cohort study was the estimation of total event rates for non-fatal overdoses that resulted in hospital admissions among patients who received OAT in primary care in England. We hypothesised that buprenorphine acts protectively against overdoses compared to methadone during treatment initiation, cessation, and the remaining time in or out of treatment, considering repeated overdoses over time, across the whole observation period. We also estimated hazard ratios of non-fatal overdose and we tested whether buprenorphine would have a stronger protective effect than methadone against incident overdoses during the above-mentioned risk periods.

## Methods

### Study design and data sources

We conducted a retrospective cohort study utilising patient-level, longitudinal data from general practices in England in the Clinical Practice Research Datalink (CPRD) GOLD and Aurum databases.^15^^,^^16^ The CPRD consists of anonymised primary care electronic health records, for more than 19% of UK's population. Both databases were pooled to enable delineation of a single study cohort (see Appendix, S1). The primary care cohort was interlinked to: (i) emergency department attendance data and admitted patient care data (APC) from the Hospital Episode Statistics (HES); (ii) cause-specific mortality data from the Office for National Statistics (ONS); and iii) patient neighbourhood and practice locality Index of Multiple Deprivation (IMD) quintiles - 2015 English Index.

This study was approved by the Medicines and Healthcare products Regulatory Agency (UK) Independent Scientific Advisory Committee (protocol 20_000077). The data pertaining to individual patients was collected routinely by the National Health Service (NHS), and therefore explicit consent was not required. Patients can withhold their records from inclusion in CPRD-based studies by refusing to share their personal data for research purposes, although very few do so.

### Cohort creation

The observation period commenced on date Jan 1, 1998 and ended on Dec 31, 2017. Cohort members were aged 18-64 years recorded by CPRD and had at least one month of continuous registration with their general practice before the date of their first recorded methadone or buprenorphine prescription - henceforth referred to as the ‘index date’ (Figures S1-S3). Product code lists included all available formulations of methadone and buprenorphine that are prescribed in the UK. However, the coding lists were restricted to ensure exclusion of formulations prescribed for analgesia. Thus, transdermal patches of buprenorphine, methadone linctus formulations, and some methadone injections were excluded (Table S1), in accordance with UK guidelines.^12^ In addition, sublingual tablets of buprenorphine with less than 2mg of active substance were excluded, as those products also are prescribed for pain management (Figure S4). We censored the observation period of patients who received both methadone and buprenorphine at the point of medication switch to enable propensity score calculation. To define the exit date and also account for death registration delays,^17^ the observation period was censored to include recorded deaths until Dec 31, 2017, although mortality data were available until May 31, 2019. Read and SNOMED codes from primary care records and from International Classification of Diseases Revision 10^th^ (ICD-10) codes from HES records were applied to classify ethnicity. Figure S5 illustrates the steps that were taken in extracting and manipulating the interlinked CPRD data sources.

### Exposure measures

We assumed that patients had initiated treatment on each prescription's issue date. We defined treatment duration on the basis of the issue date and by extending it by 14 days because, in England, methadone and buprenorphine prescriptions for OAT are issued via FP10MDA scripts with treatment duration that is not permitted to exceed 14 days. Treatment episodes were defined as periods of continuous treatment if the gap between the expected expiry date of the one prescription and the issue date of the next one was less than 14 days. Where this gap was more than 14 days, this was deemed to be a period of supply discontinuation and was flagged as an out-of-treatment period. We considered overlapping and duplicated prescriptions as one or more prescriptions that were issued either before the end of the first prescription's expiry date or were issued at the same date. In this scenario, the estimated expiry date was extended, and such prescriptions were presumed to be dispensed in instalments (Figure S6).

### Overdose ascertainment

We identified fatal overdoses using the standard set of ICD-9 and ICD-10 codes that are applied by the ONS to delineate drug poisoning deaths (Table S2). For non-fatal overdose, there is no consensus in the literature in terms of applied codes and fields of secondary care data (Appendix S1). ICD-10 codes were applied to ascertain non-fatal overdoses from relevant hospital discharge diagnoses in the APC HES data (Table S3) and were reviewed by co-investigators TM and RTW. History of overdose increases the likelihood of experiencing subsequent overdoses^2^ and therefore history of overdose may act as a strong effect modifier of subsequent overdose risk. In addition to the ICD-10 codes, we developed a list of Read and SNOMED codes to identify overdose history from primary care records (Table S4).

### Statistical analysis

We estimated the rate ratio for patients who experienced multiple non-fatal events by applying Poisson, or negative binomial regression models if overdispersion was evident, using the rate of non-fatal overdoses as the examined outcome.

To minimise confounding by indication, methadone and buprenorphine recipients were weighted according to baseline characteristics by applying inverse probability weights (IPWs). First, propensity scores were generated on the probability of being prescribed methadone versus buprenorphine and then the inverse probability was calculated to estimate stabilised IPWs. Age at baseline and gender, socioeconomic position^18^ (using patient neighbourhood and, if missing, practice locality IMD quintiles), ethnicity, region, history of overdose or self-harm,^2^ severe mental illness diagnosis,^19^ alcohol misuse,^20^ use of antipsychotics or benzodiazepines,^21^ in the year before the index date all may act as potential confounders. These variables were used to balance baseline characteristics and generate a pseudo-population that was weighted using IPWs. As a secondary analysis, we calculated crude incidence rates and estimated adjusted and weighted hazard ratios (HRs). To generate HRs, patients who experienced an overdose were censored at the event date. We fitted time-fixed variables (e.g. gender, physical comorbidities, severe mental illness at baseline) and time-varying variables (overdose/self-harm history, age and time-period of overdose) as potential confounders in multivariable models. For time-varying estimates, stratification and interaction terms with time were applied or the HR was calculated for discrete time intervals.

### Sensitivity analyses

We assessed whether applying different periods of treatment exposure and discontinuation changed the results materially. Instead of a 14-day assumed duration for each prescription, a 7-day interval was applied. Similarly, assumed treatment discontinuation duration of 7 days was applied to examine the degree to which a different classification of ‘out-of-treatment’ periods changed the results. In addition, we censored the observation period one year after the completion of the last treatment episode. Quantitative bias analysis was applied using the E-value methodology^22^ to postulate the magnitude of unobserved confounding that would need to exist to fully account for the observed association between exposure and outcome.

All analyses were performed using R version 4.0.2.

### Role of the funding source

The funder of the study had no role in study design, data collection, data analysis, data interpretation, or writing of the report.

## Results

The cohort consisted of 20898 patients who received treatment for opioid use disorder and who were observed over 83856 person-years in aggregate, of whom 73% received methadone and the remainder buprenorphine. The median length of observation period per patient was 2·3 years with interquartile range (IQR) 0·76-5·96. Table 1 summarises the characteristics of participants at baseline by comparing methadone and buprenorphine recipients, including the absolute standardised difference from the IPWs and the chi-squared test findings for covariates that were not included in the propensity score calculations.

**Table 1:** Baseline characteristics of methadone and buprenorphine recipients at start of the study's observation period as recorded in the combined CPRD GOLD and Aurum dataset.

	Methadone (n %)	Buprenorphine (n %)	*p*-value	ASD weighted
**Patients**	15155	5743		
**Region (%)**			<0·001	
South West	4092 (27·0)	1621 (28·2)		−0·003
North West	2750 (18·1)	751 (13·1)		0·010
West Midlands	2313 (15·3)	976 (17·0)		−0·007
London	1944 (12·8)	710 (12·4)		0·000
North East	1169 (7·7)	244 (4·2)		
South Central	1050 (6·9)	613 (10·7)		−0·003
Yorkshire & The Humber	704 (4·6)	262 (4·6)		0·001
East of England	611 (4·0)	212 (3·7)		−0·007
South East Coast	256 (1·7)	183 (3·2)		−0·001
East Midlands	265 (1·7)	169 (2·9)		−0·006
**Gender (%)**			<0·001	−0·007
Male	10504 (69·3)	4147 (72·2)		
Female	4651 (30·7)	1596 (27·8)		
**Age: median (IQR)**	34 (28-41)	35 (28-42)	<0·001	
**Age groups at index date (%)**			0·001	
18-24	2003 (13·2)	724 (12·6)		
25-34	5975 (39·4)	2121 (36·9)		0·006
35-44	4638 (30·6)	1875 (32·6)		−0·007
45-64	2539 (16·8)	1023 (17·8)		−0·010
**Index of Multiple Deprivation**^a^**(%)**			<0·001	
1 (least deprived)	778 (5·1)	407 (7·1)		−0·003
2	1226 (8·1)	618 (10·8)		−0·001
3	2061 (13·6)	883 (15·4)		−0·006
4	3720 (24·5)	1446 (25·2)		0·000
5 (most deprived)	7358 (48·6)	2376 (41·4)		0·007
Unknown	12 (0·1)	13 (0·2)		
**Ethnicity (%)**			<0·001	
White	12247 (80·8)	4625 (80·5)		−0·005
Asian	296 (2·0)	172 (3·0)		
Black	211 (1·4)	141 (2·5)		0·001
Mixed	136 (0·9)	66 (1·1)		−0·002
Other	88 (0·6)	38 (0·7)		0·002
Unknown	2177 (14·4)	701 (12·2)		0·006
**Treatment episodes**	40086	11568	<0·001	
Number of episodes: median (IQR)	2 (1-5)	2 (1-3)		
Days in treatment	632 (146-1806)	284 (57-905)		
Days out of treatment	803 (226-1944)	621 (150-1643)		
Days in treatment per episode	49 (14-188)	42 (14-149)		
Days out per episode	42 (19-236)	49 (17-359)		
**Days in treatment per episode (%)**			<0·001	
up to 1 month	16387 (40·9)	5104 (44·1)		
1-3 months	8435 (21·0)	2556 (22·1)		
3-6 months	4925 (12·3)	1366 (11·8)		
6-12 months	4200 (10·5)	1090 (9·4)		
> 12 months	6139 (15·3)	1452 (12·6)		
**Medication in the year before index date (%)**				
Antidepressants	4597 (30·3)	2103 (36·6)	<0·001	−0·008
Benzodiazepines	3505 (23·1)	1281 (22·3)	0·213	0·001
Z-drugs	1731 (11·4)	844 (14·7)	<0·001	
Antipsychotics	1361 (9·0)	523 (9·1)	0·797	−0·004
Gabapentinoids	650 (4·3)	305 (5·3)	0·002	−0·010
Mood stabilisers	387 (2·6)	169 (2·9)	0·130	
**Mental illness at baseline (%)**				
Alcohol dependence	3264 (21·5)	1375 (23·9)	<0·001	0·002
Depression	3179 (21·0)	1447 (25·2)	<0·001	
Anxiety disorders	2032 (13·4)	953 (16·6)	<0·001	
Schizophrenia spectrum disorders	444 (2·9)	176 (3·1)	0·640	−0·004
Personality disorders	428 (2·8)	182 (3·2)	0·202	0·000
Bipolar disorder	167 (1·1)	79 (1·4)	0·117	0·000
**Physical disease at baseline (%)**				
Asthma	1737 (11·5)	696 (12·1)	0·194	
Chronic obstructive pulmonary disease	377 (2·5)	120 (2·1)	0·102	
Pulmonary embolism	639 (4·2)	142 (2·5)	<0·001	
Hypertension	323 (2·1)	183 (3·2)	<0·001	
Other cardiovascular disease	256 (1·7)	86 (1·5)	0·361	
Endocarditis	90 (0·6)	20 (0·3)	0·037	
Stroke	89 (0·6)	30 (0·5)	0·650	
Acute coronary syndrome	41 (0·3)	19 (0·3)	0·560	
Ischaemic heart disease	41 (0·3)	19 (0·3)	0·560	
Heart failure	39 (0·3)	7 (0·1)	0·089	
Myocardial infarction	38 (0·3)	12 (0·2)	0·694	
Angina	20 (0·1)	8 (0·1)	1·000	
Arrhythmia	11 (0·1)	5 (0·1)	0·954	
Chronic liver disease	353 (2·3)	102 (1·8)	0·017	
Chronic kidney disease	143 (0·9)	54 (0·9)	1·000	
Diabetes (Type 1 or 2)	200 (1·3)	84 (1·5)	0·465	
Gastric ulcer	257 (1·7)	116 (2·0)	0·128	
Hepatitis B	809 (5·3)	180 (3·1)	<0·001	
Hepatitis C	325 (2·1)	67 (1·2)	<0·001	
Other hepatitis	64 (0·4)	10 (0·2)	0·010	
HIV	61 (0·4)	11 (0·2)	0·028	

ASD: Adjusted standardised difference; IQR: Interquartile range; HIV: Human Immunodeficiency Virus; *P-values calculated using the chi-squared test.

a Combination of patient neighbourhood (more than 99%) and practice level Index of Multiple Deprivation data (less than 1%) have been applied.

Data for 16240 (78%) patients and for 4658 (22%) patients were extracted from the CPRD Aurum and GOLD databases, respectively. Seventy percent (14651) of the patients who received OAT were males, 72% of whom were methadone recipients. Tables S5-S6 show the most frequently prescribed OAT product codes and formulations. The median age at cohort entry was 34 (IQR 28-41) years for methadone and 35 (IQR 28-42) for buprenorphine. Forty seven percent of patients lived in neighbourhoods in the most deprived IMD quintile, more than 22% were prescribed benzodiazepines and more than 30% antidepressants in the year before index date. Methadone patients had a slightly longer period of continuous treatment (median: 49 days; IQR 14-188) compared to buprenorphine patients (median: 42 days; IQR 14-149) whereas 40·9% and 44·1% of methadone and buprenorphine recipients, respectively, had treatment episodes with duration shorter than one month.

During the observation period, the incidence rate of non-fatal overdoses was 6·71 per 100 person-years, there were 12973 hospital admissions for overdoses, including multiple events per patient (event rate: 15·47 per 100 person-years) and 3219 patients switched treatment modality. Censoring of their observation period resulted in exclusion of 623 non-fatal overdoses. More than a fifth (4512, 22%) of patients experienced at least one non-fatal overdose (Figure S7) and 217 (1%) died from overdose within a year after the completion of their last prescription (overdose death rate: 0·40 per 100 person-years). Among patients who died, 183 (84%) were methadone recipients.

Table 2 contains incidence rate ratios (IRRs) generated from negative binomial regression modelling of multiple overdose events. Weeks 1-4 of treatment initiation (IRR 5·59; 95% CI 5·31, 5·89) and following treatment cessation (13·39; 12·78, 14·03) appeared to be periods of greatest overdose risk elevation compared to the remaining time in treatment. In Table 3, buprenorphine's overdose risk was lower compared to methadone across all observation periods.

**Table 2:** Event rates and estimates of non-fatal overdoses from unadjusted, adjusted and weighted negative binomial models, stratified by opioid agonist treatment status and treatment periods.

Treatment status	Person-years	Non-fatal overdoses	Event rate^a^	RR (95% CI)	uRR (95% CI)	aRR (95% CI)	wRR (95% CI)
in	25206	3930	15·6	1 (Ref)	1 (Ref)	1 (Ref)	1 (Ref)
out	33170	7478	22·5	1·45 (1·39-1·50)	1·55 (1·43-1·69)	1·61 (1·48-1·75)	1·51 (1·42-1·60)
**Treatment period**							
in (1-4 weeks)	3598	1861	51·7	5·40 (5·07-5·75)	5·39 (5·05-5·75)	5·10 (4·78-5·45)	5·59 (5·31-5·89)
in (> 4 weeks)	21608	2069	9·58	1 (Ref)	1 (Ref)	1 (Ref)	1 (Ref)
out (1-4 weeks)	3492	3639	104·0	10·88 (10·31-11·49)	12·98 (12·25-13·77)	12·63 (11·91-13·39)	13·39 (12·78-14·03)
out (>4 weeks)	29677	3839	12·9	1·35 (1·12-1·43)	1·37 (1·29-1·46)	1·60 (1·50-1·70)	1·36 (1·30-1·43)

a per 100 person-years of follow-up; RR: rate ratio; CI: Confidence Interval; uRRs: unadjusted rate ratios; aRRs: adjusted rate ratios; wHRs: inverse probability weighted rate ratios. Observation period was restricted up to ten years of follow-up to improve model fitting.

**Table 3:** Event rates and estimates of non-fatal overdoses from unadjusted, adjusted and weighted negative binomial models, stratified by opioid agonist treatment status, treatment periods and modality.

Treatment status	Treatment	Person-years	Non-fatal overdoses	Event rate^a^	RR (95% CI)	uRR (95% CI)	aRR (95% CI)	wRR (95% CI)
all	Methadone	64232	11360	17·7	1 (Ref)	1 (Ref)	1 (Ref)	1 (Ref)
all	Buprenorphine	19623	1613	8·2	0·46 (0·44-0·49)	0·44 (0·40-0·49)	0·39 (0·37-0·42)	0·39 (0·38-0·41)
in	Methadone	25760	4158	16·1	1 (Ref)	1 (Ref)	ref	ref
in	Buprenorphine	5560	385	6·9	0·43 (0·39-0·48)	0·43 (0·37-0·51)	0·36 (0·32-0·41)	0·37 (0·34-0·39)
out	Methadone	38472	7202	18·7	1 (Ref)	1 (Ref)	1 (Ref)	1 (Ref)
out	Buprenorphine	14063	1228	8·7	0·47 (0·44-0·50)	0·43 (0·38-0·48)	0·37 (0·34-0·40)	0·36 (0·34-0·38)
in (1-4 weeks)	Methadone	3006	1945	64·7	1 (Ref)	1 (Ref)	1 (Ref)	1 (Ref)
in (1-4 weeks)	Buprenorphine	820	178	21·7	0·34 (0·29-0·39)	0·26 (0·22-0·32)	0·26 (0·21-0·31)	0·26 (0·21-0·31)
in (> 4 weeks)	Methadone	22754	2213	9·7	1 (Ref)	1 (Ref)	1 (Ref)	1 (Ref)
in (> 4 weeks)	Buprenorphine	4740	207	4·4	0·45 (0·39-0·52)	0·26 (0·22-0·32)	0·28 (0·23-0·33)	0·28 (0·25-0·31)
out (1-4 weeks)	Methadone	3050	3609	118·3	1 (Ref)	1 (Ref)	1 (Ref)	1 (Ref)
out (1-4 weeks)	Buprenorphine	799	479	59·9	0·51 (0·46-0·56)	0·50 (0·44-0·57)	0·48 (0·42-0·54)	0·47 (0·43-0·50)
out (>4 weeks)	Methadone	35423	3593	10·1	1 (Ref)	1 (Ref)	1 (Ref)	1 (Ref)
out (>4 weeks)	Buprenorphine	13264	749	5·7	0·56 (0·51-0·60)	0·63 (0·57-0·70)	0·53 (0·48-0·58)	0·53 (0·50-0·56)

a per 100 person-years of follow-up; RR: rate ratio; CI: Confidence Interval; uRRs: unadjusted rate ratios; aRRs: adjusted rate ratios; wRRs: weighted rate ratios by applying inverse probability weights.

Crude rate ratios (RR) in Table 4 showed that, overall, patients out-of-treatment had 33% increased risk for overdose compared to patients in treatment (RR 1·33; 95% CI 1·25, 1·42). Table S7 presents results from time-stratified HRs. The HR for overdose among patients with follow-up longer than three years was elevated during treatment initiation (2·43; 1·98, 2·98) and reduced (0·86; 0·73, 1·01) after treatment cessation compared to the remaining period in treatment (Table S7). In terms of treatment modality, overdose risk for buprenorphine recipients was lower compared to methadone, both in-treatment (0·58; 0·50, 0·67) and out-of-treatment (0·59; 0·53, 0·65) as well as during treatment initiation (0·66; 0·55, 0·80) and after treatment cessation (0·52; 0·45, 0·61) (Table 5).

**Table 4:** Incidence rates of non-fatal overdose, stratified by opioid agonist treatment status and treatment periods (see Table S7 for time-stratified HRs).

Treatment status	Person-years	Non-fatal overdoses	IR	RR (95% CI)
in	21798	1602	7·4	1 (Ref)
out	27219	2665	9·8	1·33 (1·25-1·42)
**Treatment period**				
in (1-4 weeks)	2904	319	11·0	1·62 (1·43-1·82)
in (> 4 weeks)	18894	1283	6·8	1 (Ref)
out (1-4 weeks)	2641	516	19·5	2·88 (2·60-3·19)
out (>4 weeks)	24578	2149	8·7	1·28 (1·20-1·38)

IR: Incidence rate per 100 person-years of follow-up; RR: rate ratio; CI: Confidence Interval.

**Table 5:** Incidence rates and unadjusted, adjusted and weighted hazard ratios of non-fatal overdose, stratified by opioid agonist treatment status, treatment periods and modality.

Treatment status	Treatment	Person-years	Non-fatal overdoses	IR	RR (95% CI)	uHR (95% CI)	aHR (95% CI)	wHRs (95% CI)
all	Methadone	49875	3753	7·5	1 (Ref)	1 (Ref)	1 (Ref)	1 (Ref)
all	Buprenorphine	17334	759	4·4	0·58 (0·54-0·63)	0·57 (0·53-0·62)	0·58 (0·54-0·63)	0·58 (0·53-0·62)
in	Methadone	20574	1458	7·1	1 (Ref)	1 (Ref)	1 (Ref)	1 (Ref)
in	Buprenorphine	5095	225	4·4	0·62 (0·54-0·72)	0·58 (0·50-0·67)	0·58 (0·51-0·67)	0·58 (0·50-0·67)
out	Methadone	29301	2295	7·8	1 (Ref)	1 (Ref)	1 (Ref)	1 (Ref)
out	Buprenorphine	12239	534	4·4	0·56 (0·51-0·61)	0·55 (0·50-0·61)	0·60 (0·55-0·67)	0·59 (0·53-0·65)
in (1-4 weeks)	Methadone	2272	259	11·4	1 (Ref)	1 (Ref)	1 (Ref)	1 (Ref)
in (1-4 weeks)	Buprenorphine	703	63	9·0	0·79 (0·60-1·04)	0·72 (0·60-0·87)	0·67 (0·55-0·80)	0·66 (0·55-0·80)
in (> 4 weeks)	Methadone	18302	1199	6·6	1 (Ref)	1 (Ref)	1 (Ref)	1 (Ref)
in (> 4 weeks)	Buprenorphine	4392	162	3·7	0·56 (0·48-0·66)	0·47 (0·38-0·59)	0·49 (0·39-0·61)	0·49 (0·39-0·61)
out (1-4 weeks)	Methadone	2091	452	21·6	1 (Ref)	1 (Ref)	1 (Ref)	1 (Ref)
out (1-4 weeks)	Buprenorphine	633	74	11·7	0·54 (0·42-0·69)	0·54 (0·47-0·64)	0·53 (0·46-0·62)	0·52 (0·45-0·61)
out (>4 weeks)	Methadone	27210	1843	6·8	1 (Ref)	1 (Ref)	1 (Ref)	1 (Ref)
out (>4 weeks)	Buprenorphine	11606	460	4·0	0·59 (0·53-0·65)	0·62 (0·55-0·70)	0·69 (0·61-0·78)	0·69 (0·60-0·78)

IR: Incidence rate per 100 person-years of follow-up; RR: rate ratio; CI: Confidence Interval; uHRs: unadjusted hazard ratios; aHRs: adjusted hazard ratios; wHRs: weighted hazard ratios by applying inverse probability weights.

Sensitivity analyses did not alter the direction of the observed associations (Tables S8-S15). When we shortened the assumed treatment duration period from 14 to 7 days, patients in treatment had a lower risk of overdose compared to patients who remained untreated. In addition, the first four weeks during treatment initiation and cessation were associated with elevated overdose risk. Narrowing of the outcome definition to include only those overdoses that occurred within one year after the end of the last OAT prescription did not materially change the results. Finally, findings from quantitative bias analysis showed that an unmeasured confounder must be two-fold to entirely explain the observed associations between overdose and treatment modality (Table S16).

## Discussion

Among 20898 patients who were prescribed OAT in primary care, we found that the first four weeks of treatment initiation and cessation are periods of elevated risk for non-fatal overdose. Although we cannot infer causality from these observations, patients who were prescribed buprenorphine appear to be at a lower risk of non-fatal overdose compared to methadone recipients, even when accounting, as far as was possible, for potential confounding by indication. In addition, considering multiple overdoses per patient, overdose risk remained elevated during treatment initiation and following treatment cessation. However, the time-varying HRs showed that patients with longer follow-up periods had elevated risk of incident overdose during treatment initiation and decreased risk during treatment cessation. In contrast, patients who were observed for less than three years were more likely to overdose after treatment cessation.

Evidence from observational studies is sparse on non-fatal overdose risk in this patient population, as systematic reviews have focused on the prevalence of non-fatal overdose among drug users.^5^ Previous studies of non-fatal overdose reported incidence rates that varied greatly between 3·05 and 35·70 per 1000 person-years of follow-up^4^^,^^23^, ^24^, ^25^ and that variability may be associated with the country where the study was conducted, the data source (self-reported events versus routinely collected data) and definition of overdose according to the ICD-10 codes that were included. The observed increased rate ratio of non-fatal overdose during the first four weeks of OAT overall follows a similar pattern to studies that have examined fatal drug overdose.^6^

A recently conducted self-controlled case-series study by Keen et al.^21^ examined non-fatal overdoses among patients who received OAT and, revealing that patients who were exposed to OAT had a lower non-fatal overdose risk compared to unexposed patients. That study highlighted the time-dependent risk of non-fatal overdose for people who received OAT with 1.24 decrease in non-fatal overdose incidence risk from weeks 1-2 to weeks 3-4 during treatment initiation, a stabilisation for the remaining period in treatment and a possible increase for the weeks 1-4 after treatment cessation. Another cohort study^26^ applied a within-individual design and compared risk of accidental overdose between patients who received methadone / buprenorphine to patients who did not receive OAT. This study found that methadone recipients had 1·67 times elevated risk for overdose versus buprenorphine-treated patients.

Our study found an association between longer periods of buprenorphine treatment with a lower risk of repeated overdoses. However, buprenorphine's protective effect appears to decrease the longer a person remains untreated. This pattern differs from studies that focused on fatal drug overdoses that compared methadone and buprenorphine during and after treatment provision, which found a decreased risk of fatal overdose more than four weeks after treatment cessation.^7^^,^^27^ It has been observed that younger and female patients are more likely to overdose,^28^ though less likely to die from an overdose,^8^ which might partly explain the observed differences.

The differences in non-fatal overdose risk between methadone and buprenorphine may be the result of their specific mechanisms of action. Although methadone stabilises patients from withdrawal symptoms, showing improved treatment retention compared to buprenorphine, at low doses and shorter treatment periods it might be less effective. Furthermore, there is some evidence suggesting that, in the UK, suboptimal prescribing may occur more frequently than in other countries^29^ and our findings showed that treatment episodes were relatively short.

We conducted a large cohort study that was linked to secondary care, ecological deprivation measures and mortality records. Compared to randomised controlled trials, key strength of observational cohort studies in the field of substance misuse is their capacity to examine outcomes that can occur over a long follow-up period. Negative binomial regression models allowed to study repeated overdoses without censoring the observation period on the occurrence of the first event as applied previously.^21^^,^^26^ This expands our understanding pertaining to overdoses, since censoring restricts the denominator when calculating rate ratios, possibly leading to under-estimation of overdose risk. Although we accounted to some extent for residual confounding, differences pertaining to treatment modality and overdose risk might not be exclusively attributable to treatment modality but instead, mediated by treatment retention, illicit drug use, or affected by patients’ varying degrees of dependence. Buprenorphine might be prescribed for patients with mild or moderate dependence, namely those who are more likely to recover. However, quantitative bias analysis showed that a confounder must be at least two times associated with both the exposure and the outcome to entirely explain the observed association. Finally, we were unable to examine whether patients who visit a GP had less severe drug misuse problems versus those who received treatment in other healthcare settings. In the UK, patients receive treatment from primary care or drug and alcohol treatment settings, whereby the geographic location of those services may influence the decision for treatment to be provided from a specific setting. However, patients managed in primary care might differ from patients receiving treatment in other settings. For instance, if people treated in specialist settings have more severe drug misuse problems, then it follows that people treated in those settings might have a higher risk of overdose than those managed in general practice settings. Nonetheless, future research should test this hypothesis utilising data from other treatment settings.

We do, however, acknowledge several limitations. We could not access dispensing data or treatment adherence information, and we therefore cannot be confident that all patients took their medication as prescribed. Also, the process for OAT consumption (supervised/unsupervised) is not recorded in CPRD, and so we were unable to examine the effect that take-home dosages might have had on overdose risk. Although we excluded certain medications that are mainly prescribed for pain management, misclassification of treatment exposure could not be eliminated entirely. Thus, some patients may have been either misclassified as receiving OAT or been excluded from the study inadvertently as having received methadone or buprenorphine for pain relief. However, we believe that if this occurred, it more likely pertained to a small number of patients. This is because our findings about standardised mortality ratios and post-hoc analysis that examined the risk of fatal overdoses were comparable to other published studies.^7^^,^^8^ Moreover, limited dosage and treatment duration data may have influenced the delineation of the study's exposure windows, potentially leading to a degree of misclassification in the exposure categories. Some ‘noise’ may thereby have been introduced in analysing the temporality of the relationships of interest, specifically whether overdoses occurred during treatment or after it had ceased, although the sensitivity analyses that we conducted did not materially alter the observed associations. Out-of-treatment periods may have arisen due to various unmeasured scenarios including treatment discontinuation, transfer to another treatment provider, imprisonment, or return to illicit drug use. Additionally, although some patients appear to have ceased treatment, their treatment may have been transferred to other healthcare providers for which information is not captured in CPRD records. This is a generic issue that affects all previous UK studies of this topic that have been conducted using primary care electronic health records.^7^

Our findings build on existing evidence pertaining to periods of elevated and attenuated non-fatal overdose risk by expanding our knowledge of the relative benefits of being treated with methadone versus buprenorphine. There is some evidence indicating that patients who are hospitalised due to drug overdose usually do not receive the appropriate recommendations for follow-up visits in primary care, and treatment engagement after hospital discharge is also limited.^30^ This highlights the importance of secondary care as an assessment point for patients at elevated overdose risk. Considering that fatal poisoning is often associated with previous overdoses and that individual characteristics may determine the outcome of a non-fatal overdose, there may be opportunities to prevent adverse events by identifying patients at elevated overdose risk. Further research is needed examining the role of OAT dosing, medication switching, treatment retention, morbidity and polypharmacy in non-fatal overdoses.

## Contributors

ED, RTW, MJC, TM and DMA conceptualised and designed the study. ED reviewed the literature, drafted the manuscript, managed the data, generated clinical code lists and conducted the statistical analyses. MJC did the initial data extraction from the CPRD databases, advised on the statistical analyses and generated clinical code lists. ED and MJC accessed and verified the data. RTW and TM reviewed clinical code lists of fatal and non-fatal overdoses. RTW, MJC, TM and DMA supervised the study procedures. All authors critically reviewed drafts of the manuscript and provided intellectually significant input that informed its revision.

## Data sharing statement

In this study we used anonymised patient-level data from the CPRD that are not publicly available due to confidentiality considerations. However, researchers can access CPRD's databases by contacting the CPRD. Details of the application process and conditions of access are available at https://www.cprd.com/Data-access.

## Declaration of interests

ED, RTW, MJC declare no competing interests. TM has received research funding from the UK National Treatment Agency for Substance Misuse (Public Health England / Office for Health Improvement and Disparities), ESRC, NIHR, the European Research Area Network on Illicit Drugs / Department of Health, the Home Office, and Change Grow Live (a third sector, charitable, provider of substance misuse treatment services). He has been DMC Chair (no remuneration) for Extended-release Pharmacotherapy for Opioid Use Disorder (EXPO): Randomised Controlled Trial of Injectable Depot Maintenance Buprenorphine versus Standard-Of-Care Oral Maintenance Opioid Agonist/Partial Opioid Agonist Medication, with Personalised Psychosocial Intervention – King's College London/South London and Maudsley NHS Foundation Trust and DMC member (no remuneration) for Individual and Placement Support for Alcohol and Drug dependence (IPS-AD) Trial – Public Health England/Dept of Health. He is also a member of the UK Advisory Council on the Misuse of Drugs (no remuneration). DMA reports research grants from NIHR, AbbVie, Almirall, Celgene, Eli Lilly, Janssen, Novartis, UCB, and the Leo Foundation.

## Appendix Supplementary materials

Figure S1: Study design - Exposure-based cohort entry (index date) Image, application 1

Figure S2: Covariates and outcome assessment period for incident cases of non-fatal overdoses. Image, application 2

Figure S3: Covariates and outcome assessment period for repeated non-fatal overdoses. Image, application 3

Figure S4: Algorithm for the identification of opioid agonist treatment prescriptions utilising primary care data. Supp: suppositories; amps: ampules; SL: sublingual; lyophil: lyophilisate; TP: transdermal patches; OAT: opioid agonist treatment. Image, application 4

Figure S5: Data extraction diagram utilising the Clinical Practice Research Datalink GOLD and Aurum databases. Image, application 5

Figure S6: Construction of opioid agonist treatment episodes utilising prescription dates recorded in the Clinical Practice Research Datalink GOLD and Aurum databases. Image, application 6

Figure S7: Distribution of non-fatal overdoses. Image, application 7

Image, application 8

Image, application 9

Image, application 10

Image, application 11

Image, application 12

Image, application 13

Image, application 14

Image, application 15

Image, application 16

Image, application 17

Image, application 18

Image, application 19

Image, application 20

Image, application 21

Image, application 22

Image, application 23

Image, application 24
