# Supplementary material for: Non-fatal overdose risk during and after opioid agonist treatment: A primary care cohort study with linked hospitalisation and mortality records
Source: Lancet Reg Health Eur. 2022 Aug 11;22:100489. doi: 10.1016/j.lanepe.2022.100489 (PMC9399254; doi:10.1016/j.lanepe.2022.100489)
Supplement: Supplementary file 1 — Figure S1: Study design - Exposure-based cohort entry (index date) [file mmc1.docx]

**Supplementary material**

**Appendix S1: Further details regarding variable definitions and cohort delineation**

**Missing data**

Lists of Read and SNOMED codes of homelessness and imprisonment were draw­­­n up. However, only a small number of cohort members had relevant codes recorded (approximately 2% of patients had homelessness and 4·5% incarceration history recorded). Although we cannot estimate the prevalence of homelessness and incarceration history in the UK, recent estimates for Eastern Europe showed that among people who inject drugs, 21·9% (15·9–27·9) had unstable housing status and 36·0% (29·8–41·1) had recorded incarceration history.^1^ Therefore, we assumed that these two variables would have a small impact to our analyses. In addition, since it is hard to decide the temporality of the place of residence or incarceration and, given that people cycle in and out of medication, these codes do not add a significant value because absence of code does not necessarily imply absence of an event. However, it has been acknowledged that these variables may act as confounders, and we estimated the potential influence by applying the E-value methodology.

**Cohort delineation**

Practices are not selected for inclusion in the CPRD by a predesigned sampling procedure. However, the representativeness of the included practices and their registered patient populations have been evaluated, and both have been found to be broadly representative of the national distributions of age, gender and ethnicity. It has been estimated that the CPRD databases currently represent more than 19% of the UK population.^2,3^

For the specific purposes of our study, we restricted the CPRD databases according to the procedures that are explained in the ‘Methods’ section and Figures S4 and S5 to thereby delineate a cohort of patients with opioid use disorder. Our approach to identifying the cohort was similar to that used in other published CPRD-based studies of this patient population.^4,5^ The differences between the CPRD GOLD and Aurum datasets are threefold as follows:

1. Number of GP practices: 394 and 1370 practices contributing data to GOLD and Aurum, respectively (as of the January 2021 builds);
2. Geographic coverage: GOLD includes practices located across the UK (in England, Northern Ireland, Scotland and Wales) whereas Aurum includes mostly practices in England and a small number of practices in Northern Ireland;
3. Software: Vision® (GOLD practices) and EMIS Web® (Aurum practices) supply data to the CPRD through the NHS Digital.

**Details on patients who migrated between CPRD GOLD and CPRD Aurum datasets**

CPRD GOLD collects data from primary care general practices that use the Vision® software, with practices that use the EMIS Web® software contributing data to CPRD Aurum. Some practices have migrated from the Vision® to EMIS Web® software. Thus, historical data from these practices are included in both CPRD GOLD and Aurum datasets, resulting in a small percentage of overlap.^6^ This overlap was identified and removed using the bridging file that CPRD provides and patients who migrated to CPRD Aurum were retained in CPRD GOLD.

**Specifying daily dosage information: approaches taken by other research groups**

Previous studies^4,7^ have delineated OAT cohorts using CPRD by excluding pharmaceutical products based on daily dosage information. We did not take this approach for two reasons: 1) incomplete dosage information captured, which precluded further analysis; and 2) in the UK there are concerns about OAT underdosing; data suggest that patients received sub-optimal mean daily doses of methadone below the recommended 60mg.^4,7–9^ Therefore, imputing missing dosage data would erroneously exclude many OAT recipients. We also recognise that it used to be possible to access in the CPRD extra textual information that GPs may have entered in patients’ electronic health records, indicating prescriptions for pain relief, for instance. Such information is no longer available in CPRD data extractions due to information governance considerations.

**Diagnostic codes**

We examined comorbidities at baseline, utilising diagnostic codes from both primary and secondary care records. To identify baseline comorbidities from primary care records, we examined the period before index date since the date a patient was first enrolled with a GP practice. Diagnostic codes are available from online repositories (e.g.: <https://clinicalcodes.rss.mhs.man.ac.uk/>; <https://datacompass.lshtm.ac.uk/>; <https://www.qresearch.org/qcode-group-library/>; <https://www.opencodelists.org/>). In HES records, all available diagnostic fields were examined. When there was a difference between primary and secondary care diagnosis date, the earliest date was applied.

**Overdose ascertainment**

The World Health Organization prompts coders to assign X40-X49, X60-69, Y10-Y19 and complementary T36-T50 codes to overdoses that occurred among patients without recognised substance misuse disorders, and allocate F10-F19 codes for ‘acute overdose intoxication’ that occurred among patients with substance misuse disorders.^10^ However, in practice, both F and T codes are applied.^11^ Additionally, previously conducted studies ascertained non-fatal overdoses only in cases that involved opioids.^12,13^ It is expected that among patients with opioid use disorder, the majority of overdoses may involve opioids, although the specific drugs are not always recorded^14^ and multiple drugs are often involved. ICD-9 and ICD-10 codes were applied to classify as fatal overdoses those deaths that involved a relevant underlying death cause according to the ONS harmonised definition.

**Further information regarding delineation of the study cohort**

In addition to the algorithm to determine whether certain prescription referred to OAT episodes, sensitivity analyses was applied based on the ONS linkage that yielded similar findings with other studies^15,16^ in terms of crude and standardised mortality rates.

**References**

1. Degenhardt L, Peacock A, Colledge S, Leung J, Grebely J, Vickerman P, et al. Global prevalence of injecting drug use and sociodemographic characteristics and prevalence of HIV, HBV, and HCV in people who inject drugs: a multistage systematic review. Lancet Glob Heal 2017;5(12):e1192–207.

2. Clinical Practice Research Datalink. CPRD GOLD January 2021 (Version 2021.01.001) [Data set]. Clin. Pract. Res. Datalink2021;

3. Clinical Practice Research Datalink. CPRD Aurum January 2021 (Version 2021.01.001) [Data set]. Clin. Pract. Res. Datalink2021;

4. Hickman M, Steer C, Tilling K, Lim AG, Marsden J, Millar T, et al. The impact of buprenorphine and methadone on mortality: a primary care cohort study in the United Kingdom. Addiction 2018;113(8):1461–76.

5. Padmanathan P, Forbes H, Redaniel MT, Gunnell D, Lewer D, Moran P, et al. Self-harm and suicide during and after opioid agonist treatment among primary care patients in England: a cohort study. The Lancet Psychiatry 2021;0366(21):1–9.

6. Booth H, Dedman D, Wolf A. CPRD Aurum Frequently asked questions (FAQs). 2019.

7. Cornish R, Macleod J, Strang J, Vickerman P, Hickman M. Risk of death during and after opiate substitution treatment in primary care: prospective observational study in UK General Practice Research Database. BMJ 2010;341:c5475.

8. McCowan C, Kidd B, Fahey T. Factors associated with mortality in Scottish patients receiving methadone in primary care: retrospective cohort study. BMJ 2009;338:b2225.

9. Strang J, Hall W, Hickman M, Bird SM. Impact of supervision of methadone consumption on deaths related to methadone overdose (1993-2008): Analyses using OD4 index in England and Scotland. BMJ 2010;341(7774):640.

10. World Health Organization. Opioid overdose : trends, risk factors, interventions and priorities for action. 1998.

11. Di Rico R, Nambiar D, Stoové M, Dietze P. Drug overdose in the ED: A record linkage study examining emergency department ICD-10 coding practices in a cohort of people who inject drugs 11 Medical and Health Sciences 1117 Public Health and Health Services. BMC Health Serv Res 2018;18(1):945.

12. Morgan JR, Schackman BR, Weinstein ZM, Walley AY, Linas BP. Overdose following initiation of naltrexone and buprenorphine medication treatment for opioid use disorder in a United States commercially insured cohort. Drug Alcohol Depend 2019;200:34–9.

13. Kelty E, Hulse G. Fatal and non-fatal opioid overdose in opioid dependent patients treated with methadone, buprenorphine or implant naltrexone. Int J Drug Policy 2017;46:54–60.

14. Darke S, Duflou J. The toxicology of heroin-related death: estimating survival times. Addiction 2016;111(9):1607–13.

15. Lewer D, Tweed EJ, Aldridge RW, Morley KI. Causes of hospital admission and mortality among 6683 people who use heroin: A cohort study comparing relative and absolute risks. Drug Alcohol Depend 2019;204:107525.

16. Pierce M, Bird SM, Hickman M, Millar T. National record linkage study of mortality for a large cohort of opioid users ascertained by drug treatment or criminal justice sources in England, 2005-2009. Drug Alcohol Depend 2015;146:17–23.
