# Supplementary figures and images for: Non-fatal overdose risk during and after opioid agonist treatment: A primary care cohort study with linked hospitalisation and mortality records
Source: Lancet Reg Health Eur. 2022 Aug 11;22:100489. doi: 10.1016/j.lanepe.2022.100489 (PMC9399254; doi:10.1016/j.lanepe.2022.100489)

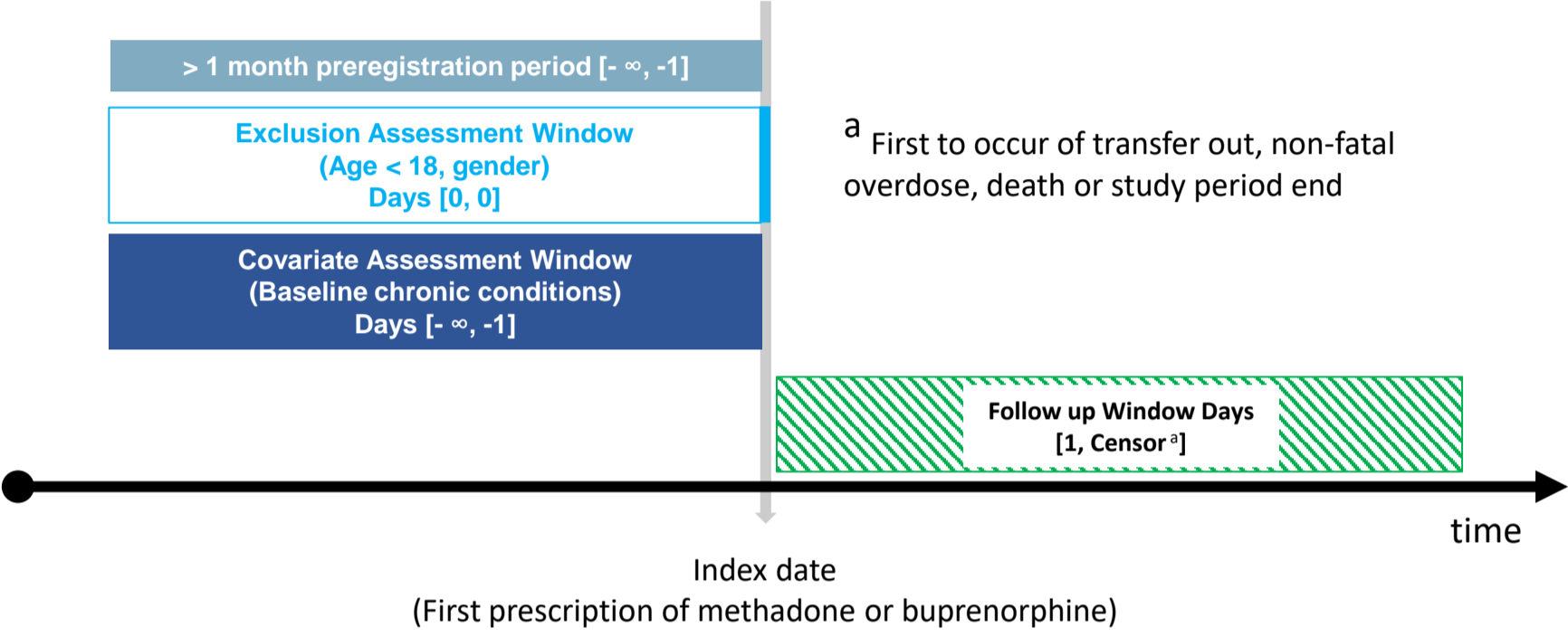

Supplement: Supplementary file 2 — Figure S2: Covariates and outcome assessment period for incident cases of non-fatal overdoses. [file mmc2.pdf]

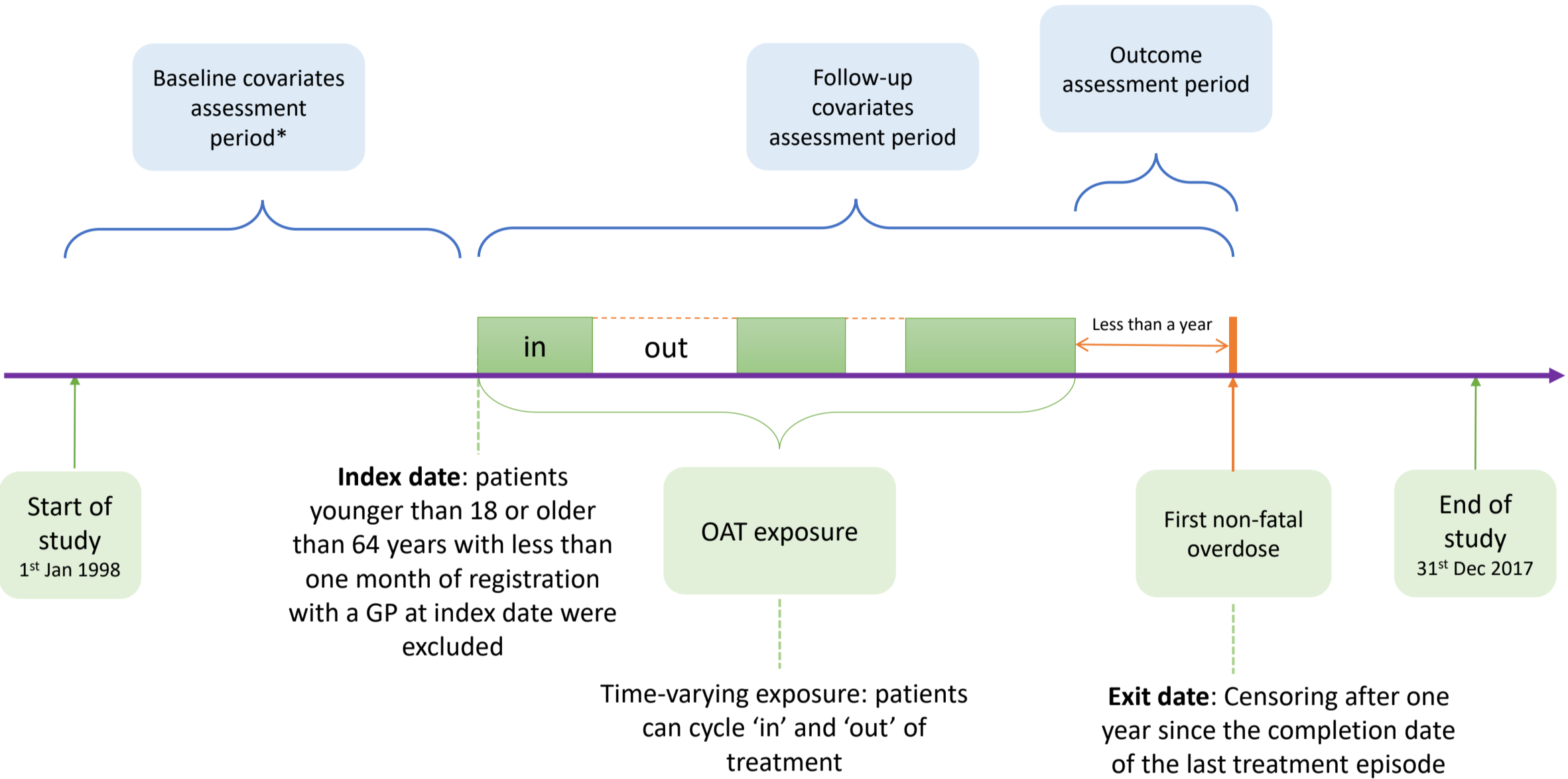

Supplement: Supplementary file 3 — Figure S3: Covariates and outcome assessment period for repeated non-fatal overdoses. [file mmc3.pdf]

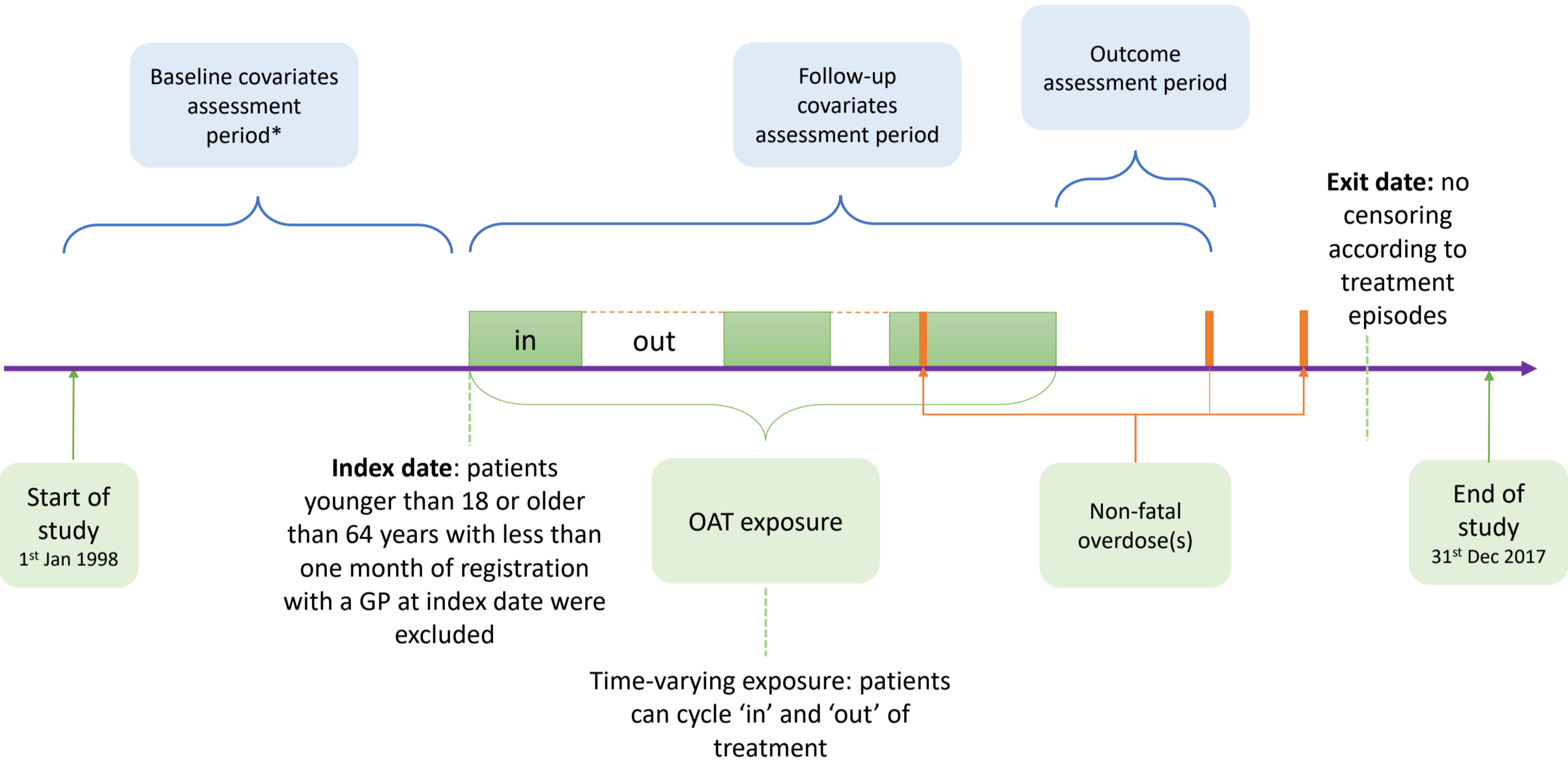

Supplement: Supplementary file 4 — Figure S4: Algorithm for the identification of opioid agonist treatment prescriptions utilising primary care data. Supp: suppositories; amps: ampules; SL: sublingual; lyophil: lyophilisate; TP: transdermal patches; OAT: opioid agonist treatment. [file mmc4.pdf]

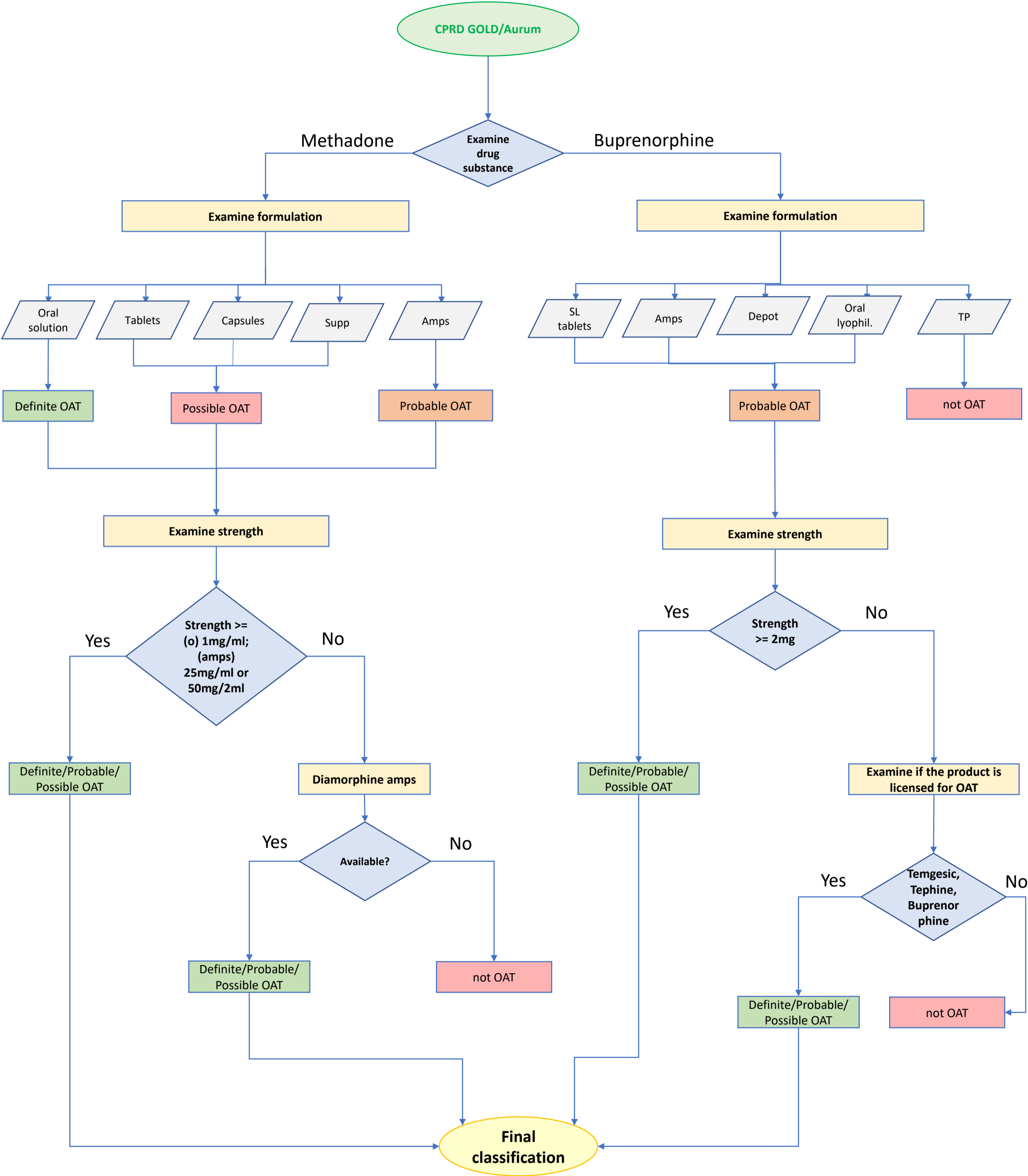

Supplement: Supplementary file 5 — Figure S5: Data extraction diagram utilising the Clinical Practice Research Datalink GOLD and Aurum databases. [file mmc5.pdf]

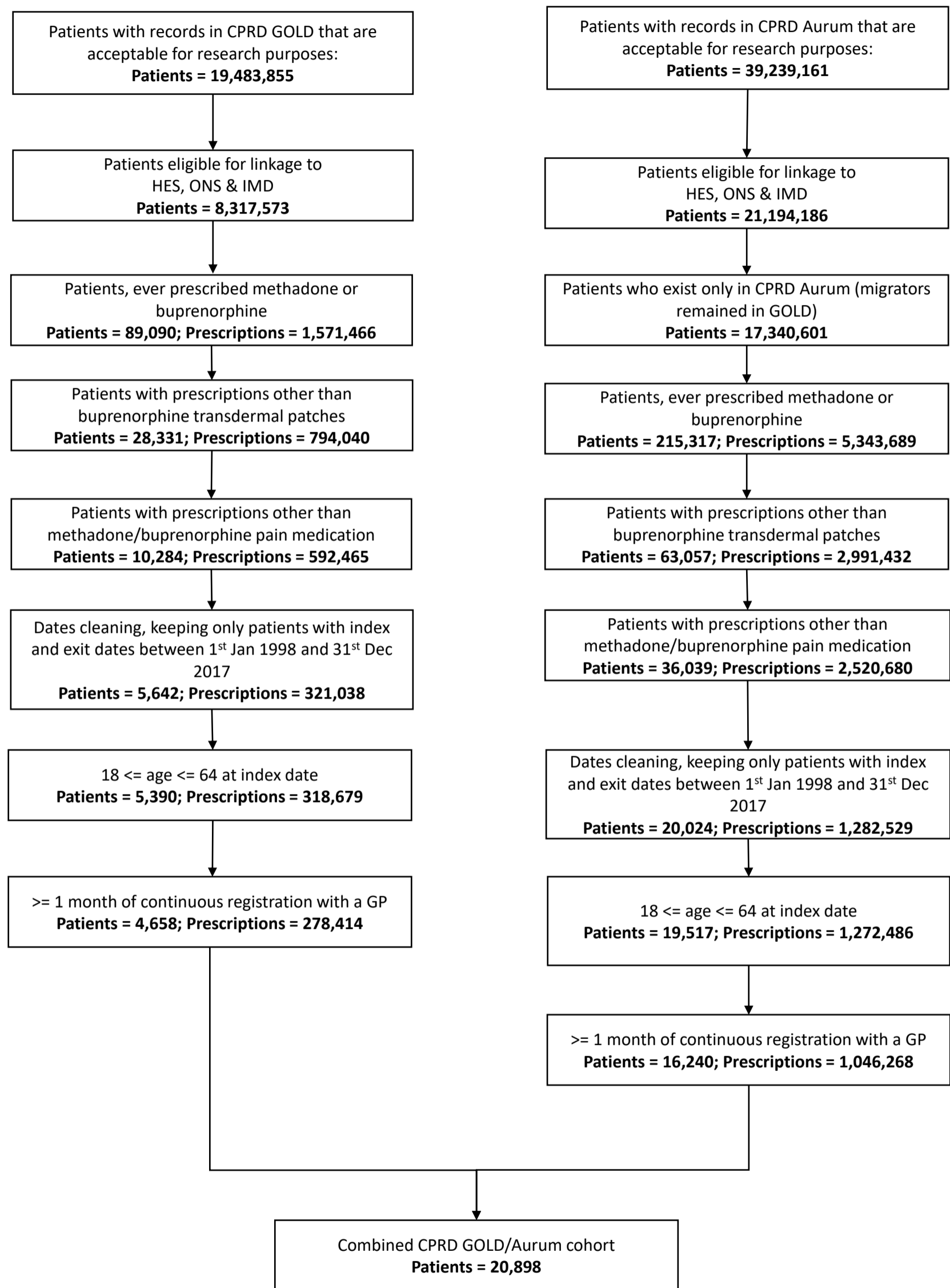

Supplement: Supplementary file 6 — Figure S6: Construction of opioid agonist treatment episodes utilising prescription dates recorded in the Clinical Practice Research Datalink GOLD and Aurum databases. [file mmc6.pdf]

Regular

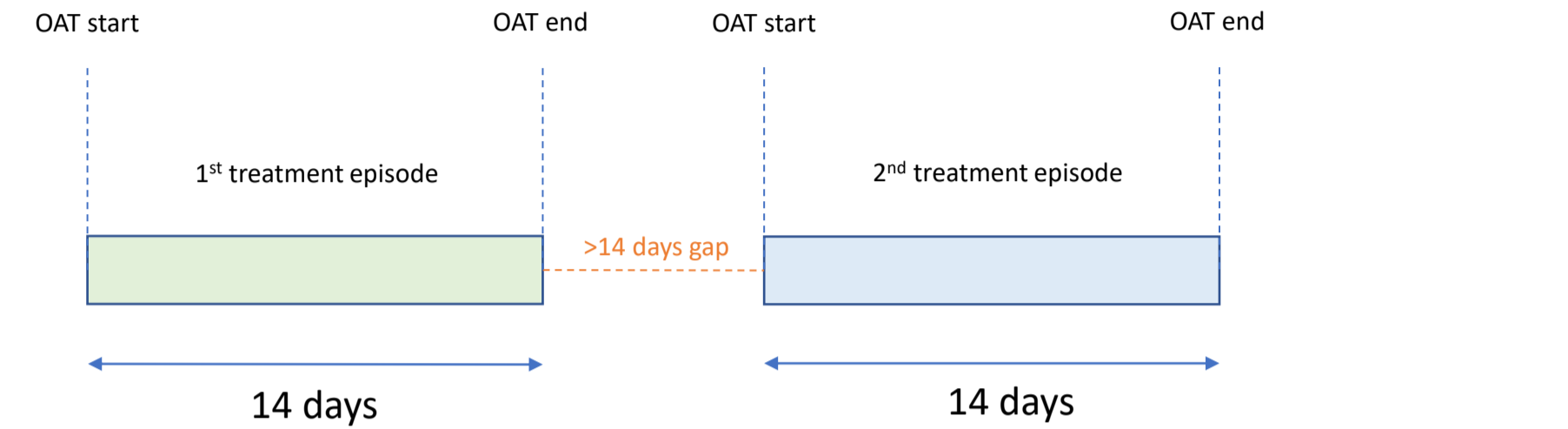

Overlapping

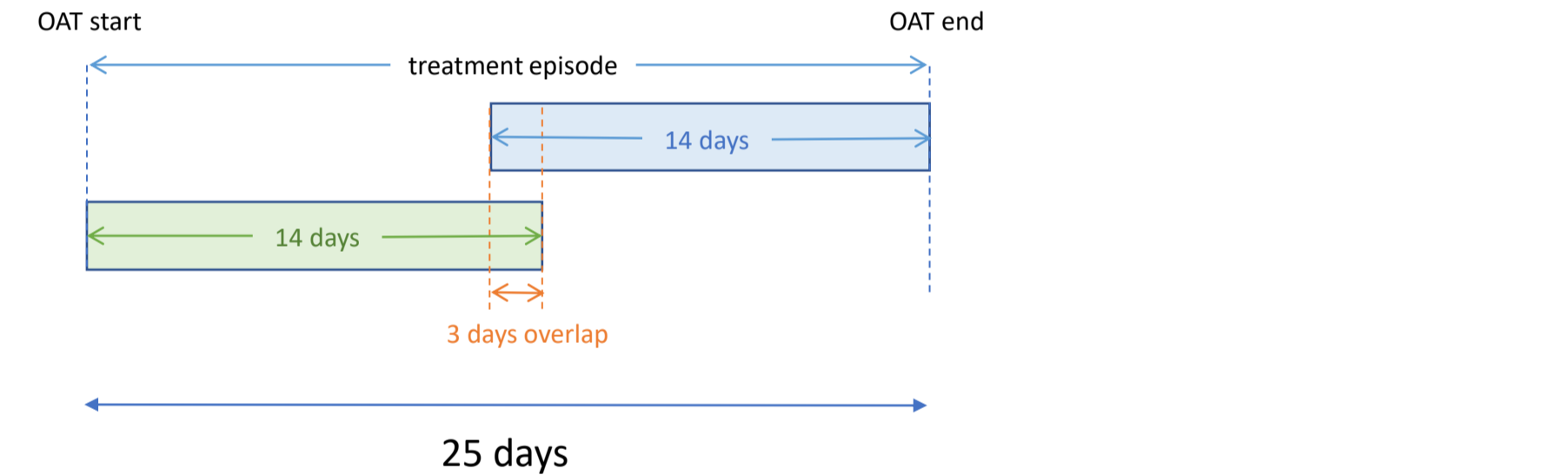

Duplicated

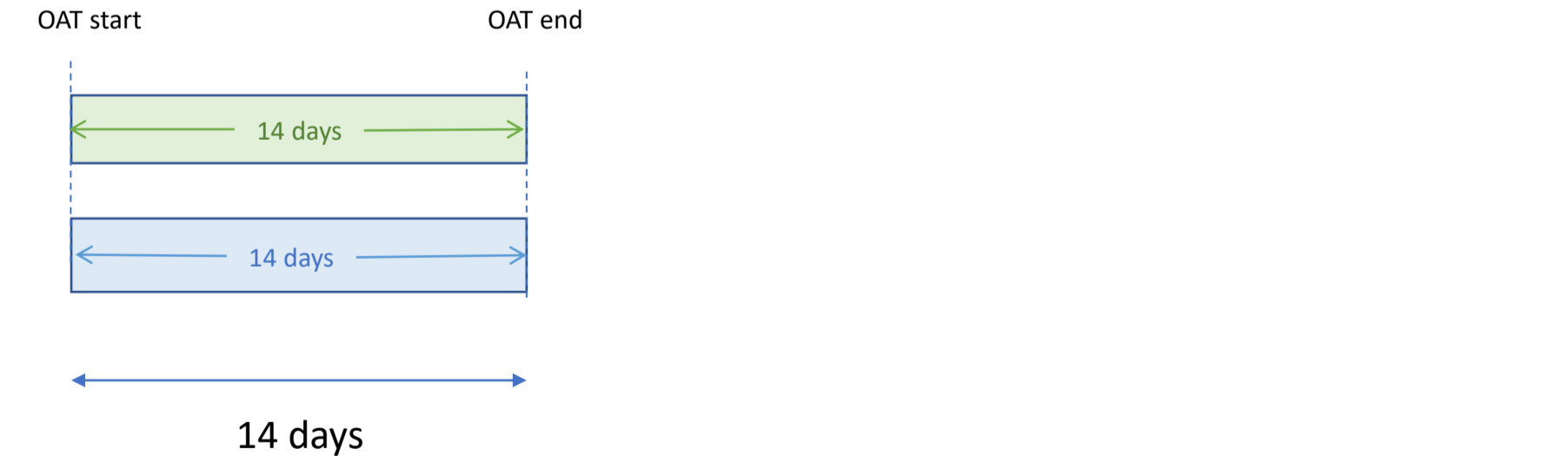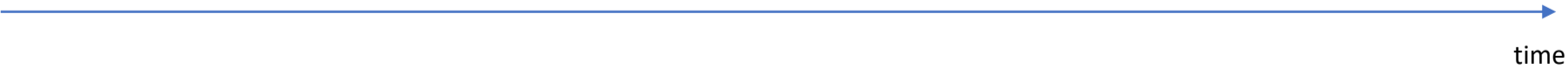

Supplement: Supplementary file 7 — Figure S7: Distribution of non-fatal overdoses. [file mmc7.pdf]

# Frequency of non-fatal overdoses

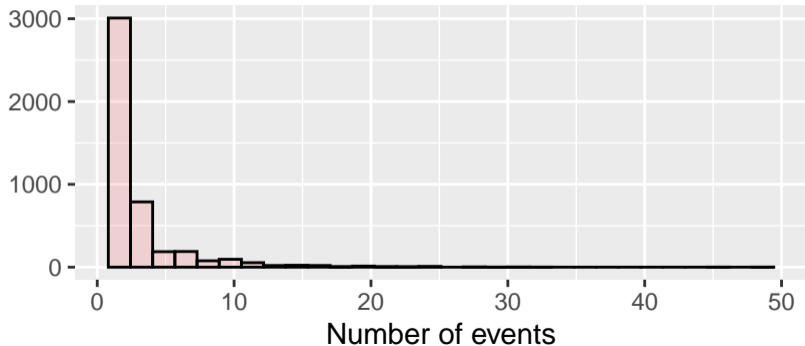

Supplement: Supplementary file 8 [file mmc8.pdf]
