# Supplementary material for: Non-fatal overdose risk during and after opioid agonist treatment: A primary care cohort study with linked hospitalisation and mortality records
Source: Lancet Reg Health Eur. 2022 Aug 11;22:100489. doi: 10.1016/j.lanepe.2022.100489 (PMC9399254; doi:10.1016/j.lanepe.2022.100489)
Supplement: Supplementary file 9 [file mmc9.docx]

**Table S1: List methadone and buprenorphine product codes in the Clinical Practice Research Datalink GOLD and Aurum datasets.**

| **Product code** | **Product name** | **Source** |
| --- | --- | --- |
| 80613 | Buprenorphine 96mg/0.27ml prolonged-release solution for injection pre-filled syringes | Gold |
| 81708 | Buvidal 96mg/0.27ml prolonged-release solution for injection pre-filled syringes (Camurus AB) | Gold |
| 81721 | Buvidal 128mg/0.36ml prolonged-release solution for injection pre-filled syringes (Camurus AB) | Gold |
| 81734 | Buvidal 16mg/0.32ml prolonged-release solution for injection pre-filled syringes (Camurus AB) | Gold |
| 81816 | Buvidal 24mg/0.48ml prolonged-release solution for injection pre-filled syringes (Camurus AB) | Gold |
| 82160 | Buvidal 8mg/0.16ml prolonged-release solution for injection pre-filled syringes (Camurus AB) | Gold |
| 68988 | Buprenorphine 8mg oral lyophilisates sugar free | Gold |
| 68989 | Buprenorphine 2mg oral lyophilisates sugar free | Gold |
| 70065 | Espranor 2mg oral lyophilisates (Martindale Pharmaceuticals Ltd) | Gold |
| 70066 | Espranor 8mg oral lyophilisates (Martindale Pharmaceuticals Ltd) | Gold |
| 6056 | Buprenorphine 8mg sublingual tablets sugar free | Gold |
| 6210 | Subutex 8mg sublingual tablets (Indivior UK Ltd) | Gold |
| 6547 | Buprenorphine 2mg sublingual tablets sugar free | Gold |
| 10077 | Subutex 2mg sublingual tablets (Indivior UK Ltd) | Gold |
| 40211 | Buprenorphine 2mg sublingual tablets sugar free (Teva UK Ltd) | Gold |
| 40212 | Buprenorphine 8mg sublingual tablets sugar free (Teva UK Ltd) | Gold |
| 50380 | Subutex 2mg sublingual tablets (Lexon (UK) Ltd) | Gold |
| 57454 | Prefibin 8mg sublingual tablets (Sandoz Ltd) | Gold |
| 58273 | Buprenorphine 2mg sublingual tablets sugar free (A A H Pharmaceuticals Ltd) | Gold |
| 59970 | Buprenorphine 2mg sublingual tablets sugar free (Actavis UK Ltd) | Gold |
| 62776 | Buprenorphine 4mg sublingual tablets sugar free | Gold |
| 62874 | Buprenorphine 6mg sublingual tablets sugar free | Gold |
| 62969 | Buprenorphine 8mg sublingual tablets sugar free (Zentiva) | Gold |
| 63640 | Subutex 2mg sublingual tablets (DE Pharmaceuticals) | Gold |
| 63788 | Buprenorphine 1mg sublingual tablets sugar free | Gold |
| 64847 | Gabup 8mg sublingual tablets (Martindale Pharmaceuticals Ltd) | Gold |
| 65157 | Buprenorphine 2mg sublingual tablets sugar free (Sigma Pharmaceuticals Plc) | Gold |
| 70464 | Subutex 2mg sublingual tablets (Waymade Healthcare Plc) | Gold |
| 71630 | Natzon 0.4mg sublingual tablets (Morningside Healthcare Ltd) | Gold |
| 71695 | Prefibin 0.4mg sublingual tablets (Sandoz Ltd) | Gold |
| 73146 | Prefibin 2mg sublingual tablets (Sandoz Ltd) | Gold |
| 80433 | Buprenorphine 2mg sublingual tablets sugar free (DE Pharmaceuticals) | Gold |
| 82575 | Subutex 8mg sublingual tablets (Waymade Healthcare Plc) | Gold |
| 35170 | Suboxone 2mg/500microgram sublingual tablets (Indivior UK Ltd) | Gold |
| 35681 | Buprenorphine 2mg / Naloxone 500microgram sublingual tablets sugar free | Gold |
| 38311 | Naloxone hc 2mg + 8mg Tablet | Gold |
| 42074 | Naloxone hc 500micrograms + 2mg Tablet | Gold |
| 26801 | Methadone colourant for Liquid | Gold |
| 33475 | Methadone 35mg/ml Injection | Gold |
| 62708 | Methadone hydrochloride powder | Gold |
| 2952 | Methadone 1mg/ml oral solution | Gold |
| 9728 | Methadone 1mg/ml oral solution sugar free | Gold |
| 11722 | Methadone 10mg/ml oral solution sugar free | Gold |
| 21562 | Physeptone 1mg/ml oral solution sugar free (Martindale Pharmaceuticals Ltd) | Gold |
| 23158 | Methadone 20mg/ml oral solution sugar free | Gold |
| 24343 | Methadose 10mg/ml oral solution concentrate (Rosemont Pharmaceuticals Ltd) | Gold |
| 24361 | Methadose 20mg/ml oral solution concentrate (Rosemont Pharmaceuticals Ltd) | Gold |
| 24440 | Methodex 1mg/ml Mixture (Link Pharmaceuticals Ltd) | Gold |
| 26277 | Physeptone 1mg/ml mixture (Martindale Pharmaceuticals Ltd) | Gold |
| 28328 | Metharose 1mg/ml oral solution sugar free (Rosemont Pharmaceuticals Ltd) | Gold |
| 29769 | Methadone 2mg/5ml Oral solution (Martindale Pharmaceuticals Ltd) | Gold |
| 30531 | Methadone 1mg/ml oral solution sugar free (Rosemont Pharmaceuticals Ltd) | Gold |
| 32237 | Methex 1mg/ml Mixture (Generics (UK) Ltd) | Gold |
| 33832 | Methadone 1mg/ml oral solution (Martindale Pharmaceuticals Ltd) | Gold |
| 36994 | Methadone 5mg/ml oral solution | Gold |
| 39437 | Eptadone 1mg/ml oral solution (Dee Pharmaceuticals Ltd) | Gold |
| 41608 | Methadone 1mg/ml oral solution (Rosemont Pharmaceuticals Ltd) | Gold |
| 41720 | Methadone 1mg/ml Mixture (Macarthy Medical Ltd) | Gold |
| 43260 | Methadone Oral solution | Gold |
| 43766 | Eptadone 5mg/ml oral solution (Dee Pharmaceuticals Ltd) | Gold |
| 47706 | Methadone 1mg/ml oral solution sugar free (Martindale Pharmaceuticals Ltd) | Gold |
| 55825 | Methadone 1mg/ml oral solution sugar free (Thornton & Ross Ltd) | Gold |
| 66921 | Methadone 1mg/ml oral solution sugar free (Waymade Healthcare Plc) | Gold |
| 68959 | Methadone 20mg/5ml oral solution | Gold |
| 69053 | Pinadone methadone 1mg/ml Oral solution sugar free (Pinewood Healthcare) | Gold |
| 70267 | Methadone 15mg/5ml oral solution | Gold |
| 76523 | Methadone 1mg/ml oral solution (Teva UK Ltd) | Gold |
| 80954 | Methadone 1mg/ml Mixture (Pinewood Healthcare) | Gold |
| 81343 | Methadone 1mg/ml oral solution sugar free (Teva UK Ltd) | Gold |
| 63077 | Methadone 1mg/5ml oral suspension | Gold |
| 17671 | Methadone 50mg/1ml solution for injection ampoules | Gold |
| 24584 | Methadone 50mg/2ml solution for injection ampoules | Gold |
| 36436 | Methadone 50mg/5ml solution for injection ampoules | Gold |
| 37507 | Physeptone 50mg/1ml solution for injection ampoules (Martindale Pharmaceuticals Ltd) | Gold |
| 46578 | Physeptone 20mg/2ml solution for injection ampoules (Martindale Pharmaceuticals Ltd) | Gold |
| 67342 | Methadone 50mg/1ml solution for injection ampoules (Alliance Healthcare (Distribution) Ltd) | Gold |
| 75719 | Physeptone 50mg/2ml solution for injection ampoules (Martindale Pharmaceuticals Ltd) | Gold |
| 35169 | Suboxone 8mg/2mg sublingual tablets (Indivior UK Ltd) | Gold |
| 35682 | Buprenorphine 8mg / Naloxone 2mg sublingual tablets sugar free | Gold |
| 68910 | Suboxone 16mg/4mg sublingual tablets (Indivior UK Ltd) | Gold |
| 70283 | Buprenorphine 16mg / Naloxone 4mg sublingual tablets sugar free | Gold |
| 71410 | Suboxone 8mg/2mg sublingual tablets (Mawdsley-Brooks & Company Ltd) | Gold |
| 25046 | Methadone diluent Liquid | Gold |
| 12132 | METHADONE 5 MG/ML INJ | Gold |
| 15449 | METHADONE 40 MG SUP | Gold |
| 21337 | METHADONE 15 MG SUP | Gold |
| 23769 | METHADONE 25 MG SUP | Gold |
| 23947 | METHADONE 30 MG SUP | Gold |
| 23948 | METHADONE 20 MG SUP | Gold |
| 24446 | METHADONE 100 MG SUP | Gold |
| 28861 | METHADONE 50 MG SUP | Gold |
| 32526 | METHADONE GREEN S/F | Gold |
| 10045241000033110 | Buprenorphine 1mg sublingual tablets sugar free | Aurum |
| 10045441000033112 | Buprenorphine 6mg sublingual tablets sugar free | Aurum |
| 10045541000033112 | Gabup 1mg sublingual tablets | Aurum |
| 10045641000033112 | Buprenorphine 4mg sublingual tablets sugar free | Aurum |
| 10045741000033116 | Gabup 0.4mg sublingual tablets | Aurum |
| 10045841000033114 | Gabup 2mg sublingual tablets | Aurum |
| 10045941000033118 | Gabup 4mg sublingual tablets | Aurum |
| 10046041000033112 | Gabup 6mg sublingual tablets | Aurum |
| 10046141000033110 | Gabup 8mg sublingual tablets | Aurum |
| 11707841000033116 | Buprenorphine 16mg / Naloxone 4mg sublingual tablets sugar free | Aurum |
| 11707941000033112 | Suboxone 16mg/4mg sublingual tablets | Aurum |
| 11789141000033120 | Buprenorphine 2mg oral lyophilisates sugar free | Aurum |
| 11789241000033114 | Buprenorphine 8mg oral lyophilisates sugar free | Aurum |
| 11789341000033116 | Espranor 2mg oral lyophilisates | Aurum |
| 11789441000033110 | Espranor 8mg oral lyophilisates | Aurum |
| 12905441000033116 | Buprenorphine 128mg/0.36ml prolonged-release solution for injection pre-filled syringes | Aurum |
| 12905541000033116 | Buprenorphine 16mg/0.32ml prolonged-release solution for injection pre-filled syringes | Aurum |
| 12905641000033120 | Buprenorphine 24mg/0.48ml prolonged-release solution for injection pre-filled syringes | Aurum |
| 12905741000033112 | Buprenorphine 32mg/0.64ml prolonged-release solution for injection pre-filled syringes | Aurum |
| 12905841000033118 | Buprenorphine 64mg/0.18ml prolonged-release solution for injection pre-filled syringes | Aurum |
| 12905941000033114 | Buprenorphine 8mg/0.16ml prolonged-release solution for injection pre-filled syringes | Aurum |
| 12906041000033116 | Buprenorphine 96mg/0.27ml prolonged-release solution for injection pre-filled syringes | Aurum |
| 12906141000033116 | Buvidal 128mg/0.36ml prolonged-release solution for injection pre-filled syringes | Aurum |
| 12906241000033112 | Buvidal 16mg/0.32ml prolonged-release solution for injection pre-filled syringes | Aurum |
| 12906341000033120 | Buvidal 24mg/0.48ml prolonged-release solution for injection pre-filled syringes | Aurum |
| 12906441000033112 | Buvidal 32mg/0.64ml prolonged-release solution for injection pre-filled syringes | Aurum |
| 12906541000033114 | Buvidal 64mg/0.18ml prolonged-release solution for injection pre-filled syringes | Aurum |
| 12906641000033110 | Buvidal 8mg/0.16ml prolonged-release solution for injection pre-filled syringes | Aurum |
| 12906741000033118 | Buvidal 96mg/0.27ml prolonged-release solution for injection pre-filled syringes | Aurum |
| 1655741000033117 | Methadose 10mg/ml oral solution concentrate | Aurum |
| 1655841000033110 | Methadose 20mg/ml oral solution concentrate | Aurum |
| 1655941000033119 | Methadone 10mg/ml oral solution sugar free | Aurum |
| 1656041000033112 | Methadone 20mg/ml oral solution sugar free | Aurum |
| 1835341000033111 | Buprenorphine 2mg sublingual tablets sugar free | Aurum |
| 1835441000033117 | Buprenorphine 8mg sublingual tablets sugar free | Aurum |
| 1835641000033115 | Subutex 2mg sublingual tablets | Aurum |
| 1835741000033112 | Subutex 8mg sublingual tablets | Aurum |
| 2003041000033115 | Methadone 1mg/ml oral solution sugar free | Aurum |
| 2069241000033110 | Physeptone 50mg/5ml solution for injection ampoules | Aurum |
| 2092941000033111 | Methadone hydrochloride powder | Aurum |
| 2103941000033113 | Metharose 1mg/ml oral solution sugar free | Aurum |
| 2659341000033116 | Methadone 50mg/2ml solution for injection ampoules | Aurum |
| 2661141000033110 | Synastone 10mg/1ml solution for injection ampoules | Aurum |
| 2661441000033119 | Synastone 50mg/5ml solution for injection ampoules | Aurum |
| 2957141000033113 | Physeptone 1mg/ml oral solution sugar free | Aurum |
| 2957241000033118 | Physeptone 1mg/ml mixture | Aurum |
| 3030641000033119 | Synastone 50mg/1ml solution for injection ampoules | Aurum |
| 3030741000033111 | Synastone 50mg/2ml solution for injection ampoules | Aurum |
| 4010641000033111 | Buprenorphine 2mg / Naloxone 500microgram sublingual tablets sugar free | Aurum |
| 4010741000033119 | Buprenorphine 8mg / Naloxone 2mg sublingual tablets sugar free | Aurum |
| 4010841000033112 | Suboxone 2mg/500microgram sublingual tablets | Aurum |
| 4010941000033116 | Suboxone 8mg/2mg sublingual tablets | Aurum |
| 4268141000033110 | Eptadone 1mg/ml oral solution | Aurum |
| 4268241000033115 | Eptadone 5mg/ml oral solution | Aurum |
| 5836641000033110 | Physeptone 50mg/1ml solution for injection ampoules | Aurum |
| 5836741000033118 | Physeptone 50mg/2ml solution for injection ampoules | Aurum |
| 6527141000033116 | Prefibin 2mg sublingual tablets | Aurum |
| 6527241000033111 | Prefibin 8mg sublingual tablets | Aurum |
| 6527441000033112 | Prefibin 0.4mg sublingual tablets | Aurum |
| 883141000033115 | Methadone 50mg/1ml solution for injection ampoules | Aurum |
| 888541000033115 | Methadone 1mg/ml oral solution | Aurum |
