# Supplementary material for: Non-fatal overdose risk during and after opioid agonist treatment: A primary care cohort study with linked hospitalisation and mortality records
Source: Lancet Reg Health Eur. 2022 Aug 11;22:100489. doi: 10.1016/j.lanepe.2022.100489 (PMC9399254; doi:10.1016/j.lanepe.2022.100489)
Supplement: Supplementary file 10 [file mmc10.docx]

**Table S2: ICD-9 and ICD-10 codes used to identify drug-poisoning deaths (according to the Office for National Statistics harmonised definition).**

| **Description** | **ICD-9 Codes** | **ICD-10 Codes** |
| --- | --- | --- |
| Mental and behavioural disorders due to psychoactive substances (excluding alcohol and tobacco) | 292, 304, 305.2–305.9 | F11–F16, F18–F19 |
| Accidental poisoning by drugs, medicaments and biological substances | E850–E858 | X40–X44 |
| Intentional self-poisoning by drugs, medicaments and biological substances | E950.0–E950.5 | X60–X64 |
| Assault by drugs, medicaments and biological substances | E962.0 | X85 |
| Poisoning by drugs, medicaments and biological substances - undetermined intent | E980.0–E980.5 | Y10–Y14 |
