# Supplementary material for: Non-fatal overdose risk during and after opioid agonist treatment: A primary care cohort study with linked hospitalisation and mortality records
Source: Lancet Reg Health Eur. 2022 Aug 11;22:100489. doi: 10.1016/j.lanepe.2022.100489 (PMC9399254; doi:10.1016/j.lanepe.2022.100489)
Supplement: Supplementary file 11 [file mmc11.docx]

**Table S3: ICD-10 codes for non-fatal overdose identification in Hospital Episode Statistics.**

| **Description** | **ICD-10 Codes** |
| --- | --- |
| Mental and behavioural disorders due to psychoactive substances (excluding alcohol and tobacco) | F11–F16, F18–F19 |
| Accidental poisoning by drugs, medicaments and biological substances | X40–X44 |
| Self-harm by drugs, medicaments and biological substances | X60–X64 |
| Poisoning by drugs, medicaments and biological substances - undetermined intent | Y10-Y14 |
| Poisoning by drugs (including opium, heroin, codeine, morphine, methadone, pethidine) | T36-T50 |
