# Supplementary material for: Non-fatal overdose risk during and after opioid agonist treatment: A primary care cohort study with linked hospitalisation and mortality records
Source: Lancet Reg Health Eur. 2022 Aug 11;22:100489. doi: 10.1016/j.lanepe.2022.100489 (PMC9399254; doi:10.1016/j.lanepe.2022.100489)
Supplement: Supplementary file 12 [file mmc12.docx]

**Table S4: List of READ, SNOMED and medical codes used to identify overdose in the Clinical Practice Research Datalink GOLD and Aurum datasets.**

| **Term** | **READ** | **SNOMED** | **Medcode Aurum** | **Medcode GOLD** |
| --- | --- | --- | --- | --- |
| Accidental poisoning by amphetamine | T842000 | 216559001 | 328655013 | 27579 |
| Accidental poisoning by analgesics,antipyretic,antirheumatic | T80..00 | 269688005 | 447191000006116 | 17930 |
| Accidental poisoning by antidepressants | T840.00 | 216545008 | 328640016 | 62986 |
| Accidental poisoning by aspirin | T803000 | 216472002 | 328555015 | 2279 |
| Accidental poisoning by cocaine | T852000 | 216583009 | 328686019 | 49910 |
| Accidental poisoning by codeine | T802000 | 216466002 | 328549013 | 51382 |
| Accidental poisoning by diazepam | T832100 | 216532009 | 328622015 | 62539 |
| Accidental poisoning by drugs acting on nervous system NOS | T85z.00 | 269688005 | 448261000006116 | 71053 |
| Accidental poisoning by drugs, medicines and biologicals | T8...00 | 269688005 | 404216015 | 414 |
| Accidental poisoning by drugs NOS | T8z..00 | 269688005 | 328735018 | 17941 |
| Accidental poisoning by gases and vapours NOS | T99z.00 | 72431002 | 328999018 | 57589 |
| Accidental poisoning by heroin | T800.00 | 216463005 | 328544015 | 54560 |
| Accidental poisoning by methadone | T801.00 | 216464004 | 328547010 | 55567 |
| Accidental poisoning by other cleaning agents | T913.00 | 72431002 | 328765011 | 28099 |
| Accidental poisoning by other drugs acting on nervous system | T85..00 | 269688005 | 449651000006113 | 34039 |
| Accidental poisoning by other non-narcotic analgesics | T807.00 | 269688005 | 328573017 | 71286 |
| Accidental poisoning by other opiates | T802.00 | 269688005 | 328548017 | 68023 |
| Accidental poisoning by paracetamol | T804100 | 290134002 | 449941000006119 | 4499 |
| Accidental poisoning by sedatives and hypnotics NOS | T82z.00 | 269688005 | 328601014 | 51632 |
| Accidental poisoning by tranquillisers | T83..00 | 216518007 | 328602019 | 37490 |
| Accidental poisoning NOS | T9z..00 | 72431002 | 329000016 | 33815 |
| Accidental poisonings | T8...99 | 269688005 | 900381000006114 | NA |
| Accidental poisonings | T9z..98 | 577691000000108 | 989881000006110 | NA |
| Accident poisoning -analgesics | T80..99 | 216462000 | 900391000006112 | NA |
| Accident poisoning - drugs NOS | T8...97 | 269688005 | 991181000006118 | NA |
| Accident poisoning - drugs NOS | T8z..99 | 643401000000101 | 900421000006116 | NA |
| Accident poisoning - NOS | T9z..99 | 577691000000108 | 900691000006116 | NA |
| Assault by poisoning by drugs or medicines | TL20.00 | 219187002 | 331607012 | 48069 |
| Cardiovascular drug poisoning | SLC..00 | 22915003 | 538151000006115 | 31074 |
| Cause of overdose - accidental | T8...11 | 269688005 | 404215016 | 1493 |
| Drug poisoning | SL...12 | 7895008 | 14060019 | 20409 |
| Heroin poisoning | SL50100 | 13187008 | 1220925010 | 20458 |
| Methadone poisoning | SL50200 | 60199004 | 498672014 | 40317 |
| Opiate and narcotic poisoning | SL50.00 | 11196001 | 1495571019 | 61673 |
| Opiate or narcotic poisoning NOS | SL50z00 | 11196001 | 324181015 | 66824 |
| Opiate poisoning | SL50.12 | 11196001 | 19399016 | 32760 |
| Other and unspecified drug and medicament poisoning | SLH..00 | 7895008 | 324438011 | 5065 |
| Poisoning | SL...00 | 75478009 | 125363016 | 19968 |
| [X]Accidental drug / other poisoning | U1A..11 | 72431002 | 361791000006111 | 20879 |
| [X]Accidental drug overdose / other poisoning | U1A..12 | 59369008 | 361801000006112 | 3390 |
| [X]Accidental poisoning by and exposure to amfetamine | U1AD.00 | 449171000000101 | 2476458016 | 42472 |
| [X]Accidental poisoning with heroin | U1A5.11 | 216463005 | 361941000006116 | 51334 |
| [X]Accidental poisoning with paracetamol | U1A0.11 | 290134002 | 361971000006112 | 49845 |
| [X]Accidental poisoning with sleeping tablets | U1A2.11 | 361149008 | 334363016 | 46445 |
| [X]Accident poisoning/exposure to narcotic drug | U1A5.00 | 449171000000101 | 361511000006113 | 48950 |
| [X]Accident poisoning/exposure to psychotropic drug | U1A4.00 | 449171000000101 | 361561000006111 | 57414 |
| [X]Accident poisoning/exposure to sedative hypnotic | U1A2.00 | 361149008 | 361571000006116 | 61559 |
| [X]Accid poison/expos to unspecif chemical at res institut | U1Ay100 | 72431002 | 361131000006111 | 107092 |
| [X]Acc poison/expos narcotic drug school/pub admin area | U1A5200 | 449171000000101 | 360021000006116 | 113119 |
| [X]Overdose - heroin | U205.11 | 295174006 | 417771000006112 | 28710 |
| [X]Poisoning by other opioids | SyuFB00 | 290173002 | 325564014 | 108575 |
| Accidental poisoning by hallucinogens | T841.00 | 216550002 | 328645014 | 68046 |
| Diphenhydramine poisoning | SL30100 | 85302003 | 1235026015 | 72451 |
| Eye drug poisoning | SLG..12 | 269271007 | 660561000006111 | 28481 |
| Unspecified opium poisoning | SL50000 | 11196001 | 324178013 | 72893 |
| [X]Accident poisoning/exposure to unspecif chemical | U1Ay.00 | 72431002 | 361581000006118 | 22839 |
