# Supplementary material for: Non-fatal overdose risk during and after opioid agonist treatment: A primary care cohort study with linked hospitalisation and mortality records
Source: Lancet Reg Health Eur. 2022 Aug 11;22:100489. doi: 10.1016/j.lanepe.2022.100489 (PMC9399254; doi:10.1016/j.lanepe.2022.100489)
Supplement: Supplementary file 13 [file mmc13.docx]

**Table S5: Summary of used product codes.**

| **Product name** | **Number of prescriptions** | **Percentage (%)** |
| --- | --- | --- |
| Methadone 1mg/ml oral solution | 549396 | 41·55 |
| Methadone 1mg/ml oral solution sugar free | 365150 | 27·61 |
| Buprenorphine 2mg sublingual tablets sugar free | 106963 | 8·09 |
| Buprenorphine 8mg sublingual tablets sugar free | 82171 | 6·21 |
| Subutex 2mg sublingual tablets (Indivior UK Ltd) | 62644 | 4·74 |
| Physeptone 1mg/ml oral solution sugar free (Martindale Pharmaceuticals Ltd) | 55725 | 4·21 |
| Subutex 8mg sublingual tablets (Indivior UK Ltd) | 47664 | 3·60 |
| Methadone Hydrochloride Oral Solution, Sugar Free, Tartrazine Free 1 mg/1 ml | 13533 | 1·02 |
| Physeptone 1mg/ml mixture (Martindale Pharmaceuticals Ltd) | 9802 | 0·74 |
| Methadone 10mg/ml oral solution sugar free | 5803 | 0·44 |
| Suboxone 2mg/500microgram sublingual tablets (Indivior UK Ltd) | 5290 | 0·40 |
| Suboxone 8mg/2mg sublingual tablets (Indivior UK Ltd) | 5113 | 0·39 |
| Buprenorphine 2mg / Naloxone 500microgram sublingual tablets sugar free | 4833 | 0·37 |
| Buprenorphine 8mg / Naloxone 2mg sublingual tablets sugar free | 3855 | 0·29 |
| Methadone 50mg/1ml solution for injection ampoules | 3066 | 0·23 |
| Methadone 1mg/ml oral solution sugar free (Thornton & Ross Ltd) | 522 | 0·04 |
| Methadone 20mg/ml oral solution sugar free | 139 | 0·01 |
| Buprenorphine 4mg sublingual tablets sugar free | 137 | 0·01 |
| Methadone Hydrochloride Diluent Oral Solution Sugar Free | 136 | 0·01 |
| Buprenorphine 6mg sublingual tablets sugar free | 135 | 0·01 |
| Methadose 10mg/ml oral solution concentrate (Rosemont Pharmaceuticals Ltd) | 101 | 0·01 |
| Buprenorphine 1mg sublingual tablets sugar free | 62 | < 0·01 |
| Methadone 50mg/2ml solution for injection ampoules | 24 | < 0·01 |
| Methadone colourant for Liquid | 17 | < 0·01 |
| Methadone 5mg/ml oral solution | 16 | < 0·01 |
| Buprenorphine 2mg oral lyophilisates sugar free | 13 | < 0·01 |
| Methadone 1mg/ml oral solution sugar free (Rosemont Pharmaceuticals Ltd) | 13 | < 0·01 |
| Metharose 1mg/ml oral solution sugar free (Rosemont Pharmaceuticals Ltd) | 8 | < 0·01 |
| Buprenorphine 8mg oral lyophilisates sugar free | 6 | < 0·01 |
| Methadose 20mg/ml oral solution concentrate (Rosemont Pharmaceuticals Ltd) | < 5 | < 0·01 |
| Prefibin 8mg sublingual tablets (Sandoz Ltd) | < 5 | < 0·01 |
| Prefibin 2mg sublingual tablets (Sandoz Ltd) | < 5 | < 0·01 |
| Methadone 1mg/ml oral solution (Martindale Pharmaceuticals Ltd) | < 5 | < 0·01 |
| Methadone diluent Liquid | < 5 | < 0·01 |
| Buprenorphine 16mg / Naloxone 4mg sublingual tablets sugar free | < 5 | < 0·01 |
| Physeptone 50mg/1ml solution for injection ampoules (Martindale Pharmaceuticals Ltd) | < 5 | < 0·01 |
| Methadone 1mg/ml Mixture (Macarthy Medical Ltd) | < 5 | < 0·01 |
| Methodex 1mg/ml Mixture (Link Pharmaceuticals Ltd) | < 5 | < 0·01 |
