# Supplementary material for: Non-fatal overdose risk during and after opioid agonist treatment: A primary care cohort study with linked hospitalisation and mortality records
Source: Lancet Reg Health Eur. 2022 Aug 11;22:100489. doi: 10.1016/j.lanepe.2022.100489 (PMC9399254; doi:10.1016/j.lanepe.2022.100489)
Supplement: Supplementary file 14 [file mmc14.docx]

**Table S6: Summary of methadone and buprenorphine formulations.**

| **Treatment** | **Formulation** | **Prescriptions** | **Percentage (%)** |
| --- | --- | --- | --- |
| Methadone | Oral solution | 1000353 | 99·69 |
| Methadone | Solution for injection | 3091 | 0·31 |
| Methadone | Liquid | 19 | <0·01 |
| Buprenorphine | Sublingual tablet | 318875 | 99·99 |
| Buprenorphine | Oral lyophilisate | 19 | 0·01 |
