# Supplementary material for: Non-fatal overdose risk during and after opioid agonist treatment: A primary care cohort study with linked hospitalisation and mortality records
Source: Lancet Reg Health Eur. 2022 Aug 11;22:100489. doi: 10.1016/j.lanepe.2022.100489 (PMC9399254; doi:10.1016/j.lanepe.2022.100489)
Supplement: Supplementary file 15 [file mmc15.docx]

**Table S7: Incidence rates and estimates from unadjusted, adjusted and weighted Cox proportional hazards models for different time-intervals.**

| **Treatment status** | **Timespan (years)** | **Person-years** | **Non-fatal overdoses** | **IR** | **RR (95% CI)** | **uHR (95% CI)** | **aHR (95% CI)** | **wHR (95% CI)** |
| --- | --- | --- | --- | --- | --- | --- | --- | --- |
| in | 0-1 | 4712 | 750 | 0·16 | 1 (1 (Ref) | 1 (Ref) | 1 (Ref) | 1 (Ref) |
| out | 0-1 | 3368 | 1077 | 0·32 | 2·01 (1·83-2·21) | 1·84 (1·67-2·04) | 1·84 (1·66-2·03) | 1·78 (1·59-2·00) |
| in | 1-3 | 6713 | 461 | 0·07 | 1 (Ref) | 1 (Ref) | 1 (Ref) | 1 (Ref) |
| out | 1-3 | 7556 | 897 | 0·12 | 1·73 (1·57-1·90) | 1·38 (1·23-1·54) | 1·37 (1·22-1·53) | 1·42 (1·25-1·62) |
| in | 3-9 | 10358 | 390 | 0·04 | 1 (Ref) | 1 (Ref) | 1 (Ref) | 1 (Ref) |
| out | 3-9 | 16283 | 691 | 0·04 | 1·13 (1·02-1·25) | 1·16 (1·03-1·31) | 1·15 (1·01-1·30) | 1·15 (1·00-1·32) |
| **Treatment period** |  |  |  |  |  |  |  |  |
| in (1-4 weeks) | 0-1 | 1880 | 256 | 0·14 | 0·78 (0·67-0·91) | 0·27 (0·16-0·47) | 0·33 (0·20-0·53) | 0·29 (0·16-0·51) |
| in (> 4 weeks) | 0-1 | 2832 | 494 | 0·17 | 1 (Ref) | 1 (Ref) | 1 (Ref) | 1 (Ref) |
| out (1-4 weeks) | 0-1 | 1461 | 328 | 0·23 | 1·29 (1·12-1·48) | 0·68 (0·43-0·89) | 0·63 (0·42-0·97) | 0·80 (0·48-1·34) |
| out (>4 weeks) | 0-1 | 1908 | 749 | 0·39 | 2·25 (2·01-2·52) | 2·05 (1·28-3·32) | 1·85 (1·14-2·47) | 2·63 (1·55-4·48) |
| in (1-4 weeks) | 1-3 | 566 | 37 | 0·07 | 0·95 (0·67-1·31) | 0·21 (0·12-0·37) | 0·20 (0·11-0·39) | 0·23 (0·12-0·44) |
| in (> 4 weeks) | 1-3 | 6147 | 424 | 0·07 | 1 (Ref) | 1 (Ref) | 1 (Ref) | 1 (Ref) |
| out (1-4 weeks) | 1-3 | 672 | 104 | 0·16 | 2·25 (1·80-2·77) | 0·40 (0·24-0·66) | 0·43 (0·25-0·78) | 0·44 (0·25-0·79) |
| out (>4 weeks) | 1-3 | 6884 | 793 | 0·12 | 1·67 (1·48-1·88) | 1·73 (1·08-2·79) | 1·79 (1·08-3·08) | 1·76 (1·04-2·97) |
| in (1-4 weeks) | >3 | 529 | 29 | 0·06 | 1·70 (1·15-2·44) | 2·60 (2·17-3·11) | 2·33 (1·95-2·81) | 2·43 (1·98-2·98) |
| in (> 4 weeks) | >3 | 13715 | 443 | 0·03 | 1 (Ref) | 1 (Ref) | 1 (Ref) | 1 (Ref) |
| out (1-4 weeks) | >3 | 590 | 94 | 0·16 | 4·94 (3·93-6·14) | 3·91 (3·31-4·62) | 3·76 (3·16-4·46) | 3·31 (2·74-4·00) |
| out (>4 weeks) | >3 | 30026 | 761 | 0·03 | 0·78 (0·70-0·88) | 0·84 (0·72-0·98) | 0·90 (0·77-1·04) | 0·86 (0·73-1·01) |

IR: incidence rate per 100 person-years of follow-up; RR: rate ratio; uHR: unadjusted hazard ratio; CI: confidence interval; aHR: adjusted hazard ratio; wHR: inverse probability weighted hazard ratios.
