# Supplementary material for: Non-fatal overdose risk during and after opioid agonist treatment: A primary care cohort study with linked hospitalisation and mortality records
Source: Lancet Reg Health Eur. 2022 Aug 11;22:100489. doi: 10.1016/j.lanepe.2022.100489 (PMC9399254; doi:10.1016/j.lanepe.2022.100489)
Supplement: Supplementary file 16 [file mmc16.docx]

**Table S8: Sensitivity analysis - alteration of treatment episodes duration from 14 to 7 days stratified by treatment status. Incidence rates and estimates from unadjusted, adjusted and weighted Cox proportional hazards models for different time-intervals.**

| **Treatment status** | **Time-span (years)** | **Person-years** | **Nonfatal overdoses** | **IR** | **RR (95% CI)** | **uHR (95% CI)** | **aHR (95% CI)** | **wHR (95% CI)** |
| --- | --- | --- | --- | --- | --- | --- | --- | --- |
| in | 0-1 | 5199 | 748 | 14·40 | 1 (Ref) | 1 (Ref) | 1 (Ref) | 1 (Ref) |
| out | 0-1 | 3630 | 1079 | 29·70 | 2·07 (1·88-2·27) | 1·58 (1·40-1·79) | 1·53 (1·35-1·73) | 1·55 (1·35-1·78) |
| in | 1-3 | 6906 | 459 | 6·65 | 1 (Ref) | 1 (Ref) | 1 (Ref) | 1 (Ref) |
| out | 1-3 | 7795 | 899 | 11·50 | 1·74 (1·55-1·94) | 1·25 (1·06-1·46) | 1·23 (1·05-1·44) | 1·25 (1·04-1·49) |
| in | 3-9 | 9583 | 390 | 4·07 | 1 (Ref) | 1 (Ref) | 1 (Ref) | 1 (Ref) |
| out | 3-9 | 16541 | 691 | 4·18 | 1·03 (0·91-1·16) | 1·00 (0·81-1·23) | 0·94 (0·76-1·16) | 0·95 (0·75-1·20) |
| **Treatment period** |  |  |  |  |  |  |  |  |
| in (1-4 weeks) | 0-1 | 2275 | 292 | 0·13 | 0·82 (0·71-0·95) | 1·08 (0·91-1·28) | 1·15 (0·97-1·37) | 1·22 (1·01-1·49) |
| in (> 4 weeks) | 0-1 | 2924 | 456 | 0·16 | 1 (Ref) | 1 (Ref) | 1 (Ref) | 1 (Ref) |
| out (1-4 weeks) | 0-1 | 1723 | 330 | 0·19 | 1·23 (1·07-1·41) | 1·87 (1·62-2·16) | 1·94 (1·68-2·24) | 1·90 (1·61-2·24) |
| out (>4 weeks) | 0-1 | 1907 | 749 | 0·39 | 2·52 (2·24-2·83) | 2·48 (2·17-2·83) | 2·29 (2·00-2·61) | 2·40 (2·06-2·78) |
| in (1-4 weeks) | 1-3 | 970 | 66 | 0·07 | 1·03 (0·79-1·33) | 0·84 (0·67-1·05) | 0·85 (0·68-1·06) | 0·82 (0·64-1·05) |
| in (> 4 weeks) | 1-3 | 5936 | 393 | 0·07 | 1 (Ref) | 1 (Ref) | 1 (Ref) | 1 (Ref) |
| out (1-4 weeks) | 1-3 | 912 | 106 | 0·12 | 1·76 (1·41-2·17) | 1·30 (1·07-1·58) | 1·31 (1·08-1·60) | 1·26 (1·01-1·68) |
| out (>4 weeks) | 1-3 | 6883 | 793 | 0·12 | 1·74 (1·54-1·97) | 1·87 (1·65-2·12) | 1·86 (1·64-2·11) | 2·04 (1·77-2·35) |
| in (1-4 weeks) | >3 | 991 | 44 | 0·04 | 1·23 (0·89-1·65) | 1·52 (1·20-1·97) | 1·15 (0·90-1·48) | 1·10 (0·85-1·44) |
| in (> 4 weeks) | >3 | 11774 | 428 | 0·04 | 1 (Ref) | 1 (Ref) | 1 (Ref) | 1 (Ref) |
| out (1-4 weeks) | >3 | 888 | 94 | 0·11 | 2·92 (2·32-3·63) | 2·52 (2·04-3·11) | 1·93 (1·56-2·39) | 1·79 (1·42-2·25) |
| out (>4 weeks) | >3 | 30026 | 761 | 0·03 | 0·70 (0·62-0·79) | 0·77 (0·68-0·87) | 1·10 (0·97-1·25) | 1·11 (0·96-1·28) |

IR: incidence rate per 100 person-years of follow-up; RR: rate ratio; uHR: unadjusted hazard ratio; CI: confidence interval; aHR: adjusted hazard ratio; wHR: inverse probability weighted hazard ratios.
