# Supplementary material for: Non-fatal overdose risk during and after opioid agonist treatment: A primary care cohort study with linked hospitalisation and mortality records
Source: Lancet Reg Health Eur. 2022 Aug 11;22:100489. doi: 10.1016/j.lanepe.2022.100489 (PMC9399254; doi:10.1016/j.lanepe.2022.100489)
Supplement: Supplementary file 17 [file mmc17.docx]

**Table S9: Sensitivity analysis - alteration of treatment episodes duration from 14 to 7 days stratified by treatment status and modality· Incidence rates and estimates from unadjusted, adjusted and weighted Cox proportional hazards models for different time-intervals.**

| **Treatment status** | **Treatment** | **Person-years** | **Non-fatal overdoses** | **IR** | **RR (95% CI)** | **uHR (95% CI)** | **aHR (95% CI)** | **wHR (95% CI)** |
| --- | --- | --- | --- | --- | --- | --- | --- | --- |
| all | Methadone | 49875 | 3753 | 7·5 | 1 (Ref) | 1 (Ref) | 1 (Ref) | 1 (Ref) |
| all | Buprenorphine | 17334 | 759 | 4·4 | 0·58 (0·53-0·63) | 0·57 (0·53-0·62) | 0·65 (0·60-0·70) | 0·64 (0·59-0·69) |
| in | Methadone | 21104 | 1461 | 6·9 | 1 (Ref) | 1 (Ref) | 1 (Ref) | 1 (Ref) |
| in | Buprenorphine | 5212 | 225 | 4·3 | 0·62 (0·54-0·72) | 0·58 (0·50-0·67) | 0·58 (0·50-0·67) | 0·60 (0·52-0·69) |
| out | Methadone | 28772 | 2292 | 8·0 | 1 (Ref) | 1 (Ref) | 1 (Ref) | 1 (Ref) |
| out | Buprenorphine | 12122 | 534 | 4·4 | 0·56 (0·51-0·61) | 0·55 (0·50-0·61) | 0·59 (0·54-0·65) | 0·65 (0·59-0·72) |
| **Treatment period** |  |  |  |  |  |  |  |  |
| in (1-4 weeks) | Methadone | 1707 | 224 | 13·1 | 1 (Ref) | 1 (Ref) | 1 (Ref) | 1 (Ref) |
| in (1-4 weeks) | Buprenorphine | 578 | 55 | 9·5 | 0·73 (0·56-0·94) | 0·65 (0·51-0·83) | 0·62 (0·48-0·79) | 0·59 (0·46-0·76) |
| in (> 4 weeks) | Methadone | 19397 | 1237 | 6·4 | 1 (Ref) | 1 (Ref) | 1 (Ref) | 1 (Ref) |
| in (> 4 weeks) | Buprenorphine | 4634 | 170 | 3·7 | 0·58 (0·49-0·68) | 0·54 (0·45-0·64) | 0·58 (0·48-0·69) | 0·57 (0·48-0·69) |
| out (1-4 weeks) | Methadone | 1561 | 449 | 28·9 | 1 (Ref) | 1 (Ref) | 1 (Ref) | 1 (Ref) |
| out (1-4 weeks) | Buprenorphine | 515 | 74 | 14·4 | 0·57 (0·44-0·72) | 0·50 (0·40-0·62) | 0·50 (0·40-0·62) | 0·47 (0·37-0·58) |
| out (>4 weeks) | Methadone | 27210 | 1843 | 6·8 | 1 (Ref) | 1 (Ref) | 1 (Ref) | 1 (Ref) |
| out (>4 weeks) | Buprenorphine | 11607 | 460 | 4·0 | 0·58 (0·53-0·65) | 0·60 (0·54-0·67) | 0·65 (0·58-0·72) | 0·73 (0·65-0·81) |

IR: incidence rate per 100 person-years of follow-up; RR: rate ratio; CI: confidence interval; uHR: unadjusted hazard ratio; aHR: adjusted hazard ratio; wHR: inverse probability weighted hazard ratios; all p-values < 0·001.
