# Supplementary material for: Non-fatal overdose risk during and after opioid agonist treatment: A primary care cohort study with linked hospitalisation and mortality records
Source: Lancet Reg Health Eur. 2022 Aug 11;22:100489. doi: 10.1016/j.lanepe.2022.100489 (PMC9399254; doi:10.1016/j.lanepe.2022.100489)
Supplement: Supplementary file 18 [file mmc18.docx]

**Table S10: Sensitivity analysis - Restricting follow-up to one year after the expiry date of last prescription of last treatment episode· Incidence rates and estimates from unadjusted, adjusted and weighted Cox proportional hazards models for different time-intervals.**

| **Treatment status** | **Treatment** | **Person-years** | **Non-fatal overdoses** | **IR** | **RR (95% CI)** | **uHR (95% CI)** | **aHR (95% CI)** | **wHR (95% CI)** |
| --- | --- | --- | --- | --- | --- | --- | --- | --- |
| all | Methadone | 34277 | 2884 | 8·4 | 1 (Ref) | 1 (Ref) | 1 (Ref) | 1 (Ref) |
| all | Buprenorphine | 9866 | 509 | 5·2 | 0·61 (0·56-0·67) | 0·64 (0·58-0·70) | 0·68 (0·61-0·74) | 0·59 (0·53-0·65) |
| in | Methadone | 20575 | 1458 | 7·1 | 1 (Ref) | 1 (Ref) | 1 (Ref) | 1 (Ref) |
| in | Buprenorphine | 5095 | 225 | 4·4 | 0·62 (0·54-0·72) | 0·61 (0·53-0·70) | 0·66 (0·57-0·76) | 0·61 (0·52-0·70) |
| out | Methadone | 13702 | 1426 | 10·4 | 1 (Ref) | 1 (Ref) | 1 (Ref) | 1 (Ref) |
| out | Buprenorphine | 4771 | 284 | 6·0 | 0·57 (0·50-0·65) | 0·62 (0·54-0·71) | 0·63 (0·56-0·72) | 0·55 (0·48-0·62) |
| **Treatment period** |  |  |  |  |  |  |  |  |
| in (1-4 weeks) | Methadone | 2272 | 259 | 11·4 | 1 (Ref) | 1 (Ref) | 1 (Ref) | 1 (Ref) |
| in (1-4 weeks) | Buprenorphine | 703 | 63 | 8·9 | 0·79 (0·59-1·03) | 0·70 (0·55-0·89) | 0·67 (0·53-0·86) | 0·61 (0·47-0·78) |
| in (> 4 weeks) | Methadone | 18302 | 1199 | 6·6 | 1 (Ref) | 1 (Ref) | 1 (Ref) | 1 (Ref) |
| in (> 4 weeks) | Buprenorphine | 4392 | 162 | 3·7 | 0·56 (0·48-0·66) | 0·57 (0·48-0·68) | 0·62 (0·52-0·74) | 0·58 (0·48-0·69) |
| out (1-4 weeks) | Methadone | 2091 | 452 | 21·6 | 1 (Ref) | 1 (Ref) | 1 (Ref) | 1 (Ref) |
| out (1-4 weeks) | Buprenorphine | 633 | 74 | 11·7 | 0·54 (0·42-0·69) | 0·57 (0·46-0·70) | 0·55 (0·44-0·68) | 0·47 (0·38-0·59) |
| out (>4 weeks) | Methadone | 11612 | 974 | 8·4 | 1 (Ref) | 1 (Ref) | 1 (Ref) | 1 (Ref) |
| out (>4 weeks) | Buprenorphine | 4138 | 210 | 5·1 | 0·61 (0·52-0·70) | 0·70 (0·60-0·82) | 0·66 (0·52-0·84) | 0·61 (0·51-0·72) |

IR: incidence rate per 100 person-years of follow-up; RR: rate ratio; CI: confidence interval; uHR: unadjusted hazard ratio; aHR: adjusted hazard ratio; wHR: inverse probability weighted hazard ratios.
