# Supplementary material for: Non-fatal overdose risk during and after opioid agonist treatment: A primary care cohort study with linked hospitalisation and mortality records
Source: Lancet Reg Health Eur. 2022 Aug 11;22:100489. doi: 10.1016/j.lanepe.2022.100489 (PMC9399254; doi:10.1016/j.lanepe.2022.100489)
Supplement: Supplementary file 19 [file mmc19.docx]

**Table S11: Sensitivity analysis - Restricting follow-up to one year after the expiry date of last prescription of last treatment episode stratified by treatment status. Incidence rates and estimates from unadjusted, adjusted and weighted Cox proportional hazards models for different time-intervals.**

| **Treatment status** | **Time-span (years)** | **Person-years** | **Non-fatal overdoses** | **IR** | **RR (95% CI)** | **uHR (95% CI)** | **aHR (95% CI)** | **wHR (95% CI)** |
| --- | --- | --- | --- | --- | --- | --- | --- | --- |
| in | 0-1 | 4712 | 750 | 0·16 | 1 (Ref) | 1 (Ref) | 1 (Ref) | 1 (Ref) |
| out | 0-1 | 3368 | 1077 | 0·32 | 2·01 (1·84-2·21) | 1·82 (1·64-2·01) | 1·73 (1·57-1·92) | 1·68 (1·50-1·89) |
| in | 1-3 | 6715 | 462 | 0·07 | 1 (Ref) | 1 (Ref) | 1 (Ref) | 1 (Ref) |
| out | 1-3 | 8685 | 413 | 0·05 | 0·69 (0·61-0·79) | 1·15 (1·00-1·31) | 1·08 (0·94-1·23) | 1·09 (0·93-1·27) |
| in | 3-9 | 10358 | 390 | 0·04 | 1 (Ref) | 1 (Ref) | 1 (Ref) | 1 (Ref) |
| out | 3-9 | 5307 | 189 | 0·04 | 0·95 (0·79-1·12) | 0·99 (0·84-1·16) | 0·88 (0·74-1·03) | 0·90 (0·75-1·08) |
| **Treatment period** |  |  |  |  |  |  |  |  |
| in (1-4 weeks) | 0-1 | 1880 | 243 | 0·13 | 0·74 (0·63-0·86) | 1·17 (0·98-1·40) | 1·07 (0·98-1·25) | 1·08 (0·95-1·31) |
| in (> 4 weeks) | 0-1 | 2832 | 494 | 0·17 | 1 (Ref) | 1 (Ref) | 1 (Ref) | 1 (Ref) |
| out (1-4 weeks) | 0-1 | 1461 | 328 | 0·22 | 1·29 (1·12-1·48) | 2·00 (1·74-2·29) | 2·10 (1·94-2·31) | 2·11 (1·74-2·39) |
| out (>4 weeks) | 0-1 | 1908 | 749 | 0·39 | 2·25 (2·01-2·52) | 2·13 (1·88-2·43) | 2·11 (1·87-2·40) | 2·15 (1·68-2·33) |
| in (1-4 weeks) | 1-3 | 567 | 38 | 0·07 | 0·98 (0·69-1·34) | 0·58 (0·44-0·76) | 0·66 (0·50-0·87) | 0·64 (0·47-0·88) |
| in (> 4 weeks) | 1-3 | 6148 | 424 | 0·07 | 1 (Ref) | 1 (Ref) | 1 (Ref) | 1 (Ref) |
| out (1-4 weeks) | 1-3 | 673 | 104 | 0·15 | 2·24 (1·80-2·77) | 1·98 (1·47-2·67) | 1·97 (1·46-2·66) | 1·87 (1·34-2·61) |
| out (>4 weeks) | 1-3 | 8013 | 309 | 0·04 | 0·56 (0·48-0·65) | 0·53 (0·40-0·70) | 0·57 (0·43-0·76) | 0·57 (0·42-0·78) |
| in (1-4 weeks) | >3 | 528 | 28 | 0·05 | 1·65 (1·10-2·37) | 0·59 (0·43-0·80) | 0·77 (0·56-1·05) | 0·74 (0·52-1·04) |
| in (> 4 weeks) | >3 | 13714 | 443 | 0·03 | 1 (Ref) | 1 (Ref) | 1 (Ref) | 1 (Ref) |
| out (1-4 weeks) | >3 | 590 | 94 | 0·16 | 4·94 (3·93-6·14) | 2·22 (1·57-3·14) | 2·28 (1·61-3·22) | 2·12 (1·45-3·11) |
| out (>4 weeks) | >3 | 5830 | 126 | 0·02 | 0·67 (0·55-0·81) | 0·34 (0·24-0·48) | 0·40 (0·28-0·57) | 0·43 (0·29-0·64) |

IR: incidence rate per 100 person-years of follow-up; RR: rate ratio; CI: confidence interval; uHR: unadjusted hazard ratio; aHR: adjusted hazard ratio; wHR: inverse probability weighted hazard ratios.
