# Supplementary material for: Non-fatal overdose risk during and after opioid agonist treatment: A primary care cohort study with linked hospitalisation and mortality records
Source: Lancet Reg Health Eur. 2022 Aug 11;22:100489. doi: 10.1016/j.lanepe.2022.100489 (PMC9399254; doi:10.1016/j.lanepe.2022.100489)
Supplement: Supplementary file 20 [file mmc20.docx]

**Table S12: Sensitivity analysis by alteration of treatment episodes duration from 14 to 7 days stratified by treatment status and time-intervals. Event rates and estimates from unadjusted, adjusted and weighted negative binomial regression models.**

| **Treatment status** | **Person-years** | **Non-fatal overdoses** | **Event Rate*** | **RR (95% CI)** | **uRR (95% CI)** | **aRR (95% CI)** | **wRR (95% CI)** |
| --- | --- | --- | --- | --- | --- | --- | --- |
| in | 30387 | 4320 | 14·2 | 1 (Ref) | 1 (Ref) | 1 (Ref) | 1 (Ref) |
| out | 53468 | 8653 | 16·2 | 1·14 (1·10-1·18) | 1·44 (1·34-1·55) | 1·45 (1·35-1·57) | 1·40 (1·33-1·49) |
| **Treatment period** |  |  |  |  |  |  |  |
| in (1-4 weeks) | 1858 | 1765 | 95·0 | 10·61 (9·98-11·30) | 9·16 (8·71-9·63) | 8·28 (7·89-8·68) | 7·76 (7·47-8·06) |
| in (> 4 weeks) | 28529 | 2555 | 9·0 | 1 (Ref) | 1 (Ref) | 1 (Ref) | 1 (Ref) |
| out (1-4 weeks) | 1832 | 3539 | 193·2 | 21·57 (20·50-22·70) | 21·68 (20·67-22·74) | 18·80 (17·89-18·68) | 19·80 (19·10-20·53) |
| out (>4 weeks) | 51636 | 5114 | 9·9 | 1·11 (1·05-1·16) | 1·65 (1·57-1·74) | 1·86 (1·78-1·96) | 2·03 (1·96-2·11) |

* per 100 person-years of follow-up; RR: rate ratio; CI: confidence interval; uRR: unadjusted rate ratio; aRR: adjusted rate ratio; wRR: inverse probability weighted rate ratios; all p-values < 0·001.
