# Supplementary material for: Non-fatal overdose risk during and after opioid agonist treatment: A primary care cohort study with linked hospitalisation and mortality records
Source: Lancet Reg Health Eur. 2022 Aug 11;22:100489. doi: 10.1016/j.lanepe.2022.100489 (PMC9399254; doi:10.1016/j.lanepe.2022.100489)
Supplement: Supplementary file 21 [file mmc21.docx]

**Table S13: Sensitivity analysis by alteration of treatment episodes duration from 14 to 7 days. Event rates and estimates from unadjusted, adjusted and weighted negative binomial regression models for different treatment status time-intervals and opioid agonist treatment modality.**

| **Treatment status** | **Treatment** | **Person-years** | **Non-fatal overdoses** | **Event Rate*** | **RR (95% CI)** | **uRR (95% CI)** | **aRR (95% CI)** | **wRR (95% CI)** |
| --- | --- | --- | --- | --- | --- | --- | --- | --- |
| all | Methadone | 64232 | 11360 | 17·7 | 1 (Ref) | 1 (Ref) | 1 (Ref) | 1 (Ref) |
| all | Buprenorphine | 19623 | 1613 | 8·2 | 0·46 (0·44-0·49) | 0·45 (0·43-0·47) | 0·50 (0·48-0·52) | 0·44 (0·43-0·46) |
| in | Methadone | 24986 | 3947 | 15·8 | 1 (Ref) | 1 (Ref) | 1 (Ref) | 1 (Ref) |
| in | Buprenorphine | 5401 | 373 | 6·9 | 0·44 (0·39-0·49) | 0·37 (0·34-0·40) | 0·43 (0·40-0·47) | 0·38 (0·36-0·39) |
| out | Methadone | 39246 | 7413 | 18·9 | 1 (Ref) | 1 (Ref) | 1 (Ref) | 1 (Ref) |
| out | Buprenorphine | 14222 | 1240 | 8·7 | 0·46 (0·43-0·49) | 0·49 (0·46-0·51) | 0·54 (0·51-0·57) | 0·48 (0·46-0·50) |
| **Treatment period** |  |  |  |  |  |  |  |  |
| in (1-4 weeks) | Methadone | 1462 | 1621 | 110·9 | 1 (Ref) | 1 (Ref) | 1 (Ref) | 1 (Ref) |
| in (1-4 weeks) | Buprenorphine | 396 | 144 | 36·4 | 0·33 (0·28-0·39) | 0·28 (0·19-0·40) | 0·36 (0·25-0·52) | 0·28 (0·20-0·41) |
| in (> 4 weeks) | Methadone | 23524 | 2326 | 9·9 | 1 (Ref) | 1 (Ref) | 1 (Ref) | 1 (Ref) |
| in (> 4 weeks) | Buprenorphine | 5005 | 229 | 4·6 | 0·46 (0·40-0·53) | 0·39 (0·33-0·45) | 0·49 (0·42-0·57) | 0·45 (0·41-0·49) |
| out (1-4 weeks) | Methadone | 1525 | 3156 | 207·0 | 1 (Ref) | 1 (Ref) | 1 (Ref) | 1 (Ref) |
| out (1-4 weeks) | Buprenorphine | 307 | 383 | 124·8 | 0·60 (0·54-0·67) | 0·48 (0·37-0·62) | 0·48 (0·37-0·62) | 0·48 (0·37-0·62) |
| out (>4 weeks) | Methadone | 37721 | 4257 | 11·3 | 1 (Ref) | 1 (Ref) | 1 (Ref) | 1 (Ref) |
| out (>4 weeks) | Buprenorphine | 13915 | 857 | 6·2 | 0·55 (0·51-0·59) | 0·64 (0·59-0·70) | 0·79 (0·72-0·87) | 0·74 (0·70-0·78) |

* per 100 person-years of follow-up; RR: rate ratio; CI: confidence interval; uRR: unadjusted rate ratio; aRR: adjusted rate ratio; wRR: inverse probability weighted rate ratios; all p-values < 0·001.
