# Supplementary material for: Non-fatal overdose risk during and after opioid agonist treatment: A primary care cohort study with linked hospitalisation and mortality records
Source: Lancet Reg Health Eur. 2022 Aug 11;22:100489. doi: 10.1016/j.lanepe.2022.100489 (PMC9399254; doi:10.1016/j.lanepe.2022.100489)
Supplement: Supplementary file 23 [file mmc23.docx]

**Table S15: Sensitivity analysis - Restricting follow-up to one year after the expiry date of last prescription of last treatment episode. Event rates and estimates from unadjusted, adjusted and weighted negative binomial regression models stratified by treatment status time-period and opioid agonist therapy modality.**

| **Treatment status** | **Treatment** | **Person-years** | **Non-fatal overdoses** | **Event Rate*** | **RR (95% CI)** | **uRR (95% CI)** | **aRR (95% CI)** | **wRR (95% CI)** |
| --- | --- | --- | --- | --- | --- | --- | --- | --- |
| all | Methadone | 43531 | 9720 | 22·33 | 1 (Ref) | 1 (Ref) | 1 (Ref) | 1 (Ref) |
| all | Buprenorphine | 10866 | 1306 | 12·02 | 0·54 (0·51-0·57) | 0·44 (0·41-0·46) | 0·47 (0·45-0·50) | 0·44 (0·43-0·45) |
| in | Methadone | 25760 | 4158 | 16·14 | 1 (Ref) | 1 (Ref) | 1 (Ref) | 1 (Ref) |
| in | Buprenorphine | 5560 | 385 | 6·92 | 0·43 (0·39-0·48) | 0·37 (0·34-0·40) | 0·39 (0·36-0·42) | 0·38 (0·36-0·40) |
| out | Methadone | 17771 | 5562 | 31·30 | 1 (Ref) | 1 (Ref) | 1 (Ref) | 1 (Ref) |
| out | Buprenorphine | 5306 | 921 | 17·36 | 0·55 (0·52-0·59) | 0·48 (0·45-0·51) | 0·52 (0·49-0·56) | 0·48 (0·46-0·50) |
| **Treatment period** |  |  |  |  |  |  |  |  |
| in (1-4 weeks) | Methadone | 772 | 1583 | 205·05 | 1 (Ref) | 1 (Ref) | 1 (Ref) | 1 (Ref) |
| in (1-4 weeks) | Buprenorphine | 236 | 140 | 59·32 | 0·29 (0·24-0·34) | 0·26 (0·18-0·37) | 0·27 (0·18-0·39) | 0·26 (0·17-0·38) |
| in (> 4 weeks) | Methadone | 24988 | 2575 | 10·30 | 1 (Ref) | 1 (Ref) | 1 (Ref) | 1 (Ref) |
| in (> 4 weeks) | Buprenorphine | 5324 | 245 | 4·60 | 0·45 (0·39-0·51) | 0·49 (0·43-0·57) | 0·52 (0·45-0·60) | 0·53 (0·48-0·57) |
| out (1-4 weeks) | Methadone | 728 | 2126 | 292·03 | 1 (Ref) | 1 (Ref) | 1 (Ref) | 1 (Ref) |
| out (1-4 weeks) | Buprenorphine | 144 | 236 | 163·89 | 0·56 (0·49-0·64) | 0·35 (0·26-0·49) | 0·38 (0·28-0·52) | 0·37 (0·31-0·45) |
| out (>4 weeks) | Methadone | 17043 | 3436 | 20·16 | 1 (Ref) | 1 (Ref) | 1 (Ref) | 1 (Ref) |
| out (>4 weeks) | Buprenorphine | 5162 | 685 | 13·27 | 0·66 (0·61-0·71) | 0·78 (0·71-0·85) | 0·79 (0·72-0·87) | 0·79 (0·75-0·83) |

* per 100 person-years of follow-up; RR: rate ratio; CI: confidence interval; uRR: unadjusted rate ratio; aRR: adjusted rate ratio; wRR: inverse probability weighted rate ratios; all p-values < 0·001.
