# Supplementary material for: Non-fatal overdose risk during and after opioid agonist treatment: A primary care cohort study with linked hospitalisation and mortality records
Source: Lancet Reg Health Eur. 2022 Aug 11;22:100489. doi: 10.1016/j.lanepe.2022.100489 (PMC9399254; doi:10.1016/j.lanepe.2022.100489)
Supplement: Supplementary file 24 [file mmc24.docx]

**Table S16: Sensitivity analysis - applying the E-value methodology to account for unobserved confounding.**

| **Treatment status** | **Treatment** | **wHR (95% CI)** | **E-value** | **wRR (95% CI)** | **E-value** |
| --- | --- | --- | --- | --- | --- |
| in | OAT | 1 (Ref) | 1 (Ref) | 1 (Ref) | 1 (Ref) |
| out | OAT | 1·45 (1·28-1·65) | 1·91 (1·66-2·18) | 1·51 (1·42-1·60) | 2·39 (2·19-2·58) |
| **Treatment period** |  |  |  |  |  |
| in (1-4 weeks) | OAT | 0·98 (0·75-1·31) | 1·11 (1·00-1·74) | 5·59 (5·31-5·89) | 10·66 (10·09-11·26) |
| in (> 4 weeks) | OAT | 1 (Ref) | 1 (Ref) | 1 (Ref) | 1 (Ref) |
| out (1-4 weeks) | OAT | 1·52 (1·16-2·04) | 2·01 (1·45-2·65) | 13·39 (12·78-14·03) | 26·27 (25·05-27·55) |
| out (>4 weeks) | OAT | 1·75 (1·11-2·82) | 2·31 (1·36-3·48) | 1·36 (1·30-1·43) | 2·06 (1·92-2·21) |
| all | Methadone | 1 (Ref) | 1 (Ref) | 1 (Ref) | 1 (Ref) |
| all | Buprenorphine | 0·58 (0·53-0·62) | 2·27 (2·13-2·47) | 0·39 (0·38-0·41) | 4·57 (4·31-4·7) |
| in | Methadone | 1 (Ref) | 1 (Ref) | 1 (Ref) | 1 (Ref) |
| in | Buprenorphine | 0·58 (0·50-0·67) | 2·27 (1·97-2·61) | 0·37 (0·34-0·39) | 4·85 (4·57-5·33) |
| out | Methadone | 1 (Ref) | 1 (Ref) | 1 (Ref) | 1 (Ref) |
| out | Buprenorphine | 0·59 (0·53-0·65) | 2·24 (2·03-2·47) | 0·36 (0·34-0·38) | 5·00 (4·7-5·33) |
| in (1-4 weeks) | Methadone | 1 (Ref) | 1 (Ref) | 1 (Ref) | 1 (Ref) |
| in (1-4 weeks) | Buprenorphine | 0·66 (0·55-0·80) | 2·00 (1·61-2·39) | 0·26 (0·21-0·31) | 7·15 (5·91-8·99) |
| in (> 4 weeks) | Methadone | 1 (Ref) | 1 (Ref) | 1 (Ref) | 1 (Ref) |
| in (> 4 weeks) | Buprenorphine | 0·49 (0·39-0·61) | 2·65 (2·16-3·22) | 0·28 (0·25-0·31) | 6·60 (5·91-7·46) |
| out (1-4 weeks) | Methadone | 1 (Ref) | 1 (Ref) | 1 (Ref) | 1 (Ref) |
| out (1-4 weeks) | Buprenorphine | 0·52 (0·45-0·61) | 2·52 (2·16-2·86) | 0·47 (0·43-0·50) | 3·68 (3·41-4·08) |
| out (>4 weeks) | Methadone | 1 (Ref) | 1 (Ref) | 1 (Ref) | 1 (Ref) |
| out (>4 weeks) | Buprenorphine | 0·69 (0·60-0·78) | 1·91 (1·66-2·2) | 0·53 (0·50-0·56) | 3·18 (2·97-3·41) |

wHR: weighted hazard ratio; CI: confidence interval; wRR:inverse probability weighted rate ratios; OAT: opioid agonist treatment.

†E-value calculated based on the formula $\frac{1}{RR*}+sqrt[\frac{1}{RR*}\left( \frac{1}{RR*}-1 \right)]$, whereby RR* = $\left( 1-{0.5}^{\mathrm{sqrt}\left( \mathrm{HR} \right)} \right)/(1-{0.5}^{\mathrm{sqrt}\left( \frac{1}{\mathrm{HR}} \right)})$ for hazard ratios

‡E-value calculated based on the formula $\frac{1}{\mathrm{RR}}+sqrt[\frac{1}{\mathrm{RR}}\left( \frac{1}{\mathrm{RR}}-1 \right)]$ for rate ratios
